# Pyroptosis‐Amplified Decoy Nanovesicles Orchestrate Pyroptotic Antigen Explosion and Endogenous Immune Revitalization for Potentiating Tumour Immunotherapy

**DOI:** 10.1002/jev2.70356

**Published:** 2026-08-28

**Authors:** Geng Dou, Jiani Liu, Ran Tian, Keying Zhang, Meng Suo, Ding Zhou, Yipeng Zhang, Xuemei Liu, Xinyu Qiu, Jinxin Kuang, Zhaonan Zou, Shuchang Wang, Lu Zhao, Feng Ding, Di Wang, Congcong Guan, Xiaoyu Zhu, Dongyao Wang, Siying Liu, Tianfu Zhang, Yimin Zhao, Shiyu Liu

**Affiliations:** ^1^ State Key Laboratory of Oral & Maxillofacial Reconstruction and Regeneration National Clinical Research Center for Oral Diseases Shaanxi Key Laboratory of Stomatology Department of Oral Biology & Clinic of Oral Rare Diseases and Genetic Disease School of Stomatology The Fourth Military Medical University Xi'an Shaanxi China; ^2^ State Key Laboratory of Oral & Maxillofacial Reconstruction and Regeneration National Clinical Research Center for Oral Diseases Shaanxi International Joint Research Center for Oral Diseases Center for Tissue Engineering School of Stomatology The Fourth Military Medical University Xi'an Shaanxi China; ^3^ State Key Laboratory of Oral & Maxillofacial Reconstruction and Regeneration National Clinical Research Center for Oral Diseases Shaanxi Key Laboratory of Stomatology Digital Center School of Stomatology The Fourth Military Medical University Xi'an Shaanxi China; ^4^ School of Chemistry and Chemical Engineering Hubei Polytechnic University Huangshi Hubei China; ^5^ Department of Urology Xijing Hospital The Fourth Military Medical University Xi'an Shaanxi China; ^6^ Guangzhou Institute of Cancer Research School of Biomedical Engineering the Affiliated Cancer Hospital Guangzhou Medical University Guangzhou Guangdong China; ^7^ Jilin Provincial Key Laboratory of Tooth Development and Bone Remodeling School and Hospital of Stomatology Jilin University Changchun Jilin China; ^8^ Department of Pediatric Dentistry Liaoning Provincial Key Laboratory of Oral Diseases School and Hospital of Stomatology China Medical University Shenyang Liaoning China; ^9^ Department of Oral and Maxillofacial Surgery School of Stomatology The Fourth Military Medical University Xi'an Shaanxi China; ^10^ Department of Orthopedics Xijing Hospital The Fourth Military Medical University Xi'an Shaanxi China; ^11^ Department of Hematology Division of Life Sciences and Medicine the First Affiliated Hospital of USTC University of Science and Technology of China Hefei Anhui China

**Keywords:** checkpoint blockade, immune revitalization, nanovesicles, pyroptosis, tumour immunotherapy

## Abstract

Current advances in tumour immunotherapy remain constrained by inadequate cytotoxic T lymphocyte activation and the immunosuppressive tumour microenvironment. Innovative strategies are urgently required to enhance anti‐tumour immune potency for clinical improvements in immunotherapy‐resistant malignancies. Herein, we developed pyroptosis‐amplified decoy nanovesicles (PADVs) to synergistically dismantle tumour resistance mechanisms and rejuvenate endogenous anti‐tumour immunity. PADVs integrate checkpoint‐neutralizing capability with precise regulation of the pyroptosis molecular switch in tumour cells, thereby initiating a sequential activation cascade that enables efficient presentation of tumour‐specific antigens to the reshaped immune system. Systemically infused PADVs demonstrated dual‐targeting priority towards lymph node and deep tumour tissues, reversing immunosuppression through competitive blockade of PD‐1/PD‐L1 and SIRPα/CD47 axes while providing co‐stimulatory signals for immune reactivation. Furthermore, PADVs alleviate the epigenetic suppression of pyroptosis in tumours via cytosolic delivery of decitabine and LPS, enabling gasdermin D (GSDMD) upregulation and cleavage. This coordinated strategy reverses T cell exhaustion and triggers explosive antigen release via GSDMD‐mediated membrane perforation, initiating a self‐amplifying immune cascade involving dendritic cell activation, effector and memory T cell formation. Consequently, PADVs demonstrate excellent efficacy in suppressing tumour progression, recurrence, and metastasis in melanoma, breast cancer, and colon cancer models. This study pioneers a versatile nanoplatform that not only counteracts tumour immune evasion but also establishes durable anti‐tumour immunity, offering a transformative approach to tumour immunotherapy.

## 1 Introduction

The emergence of immunotherapy has revolutionized tumour treatment paradigms. Modalities such as immune checkpoint inhibitors (ICIs) (Waldman et al. 2020; Zhang and Zhang 2020), adoptive cell therapies (Waldman et al. 2020; Zhang and Zhang 2020) and tumour vaccines (Zhang and Zhang 2020; Yang et al. 2024) have achieved unprecedented long‐term survival in a subset of patients (Kraehenbuehl et al. 2022; Morad et al. 2021). Nevertheless, the broader clinical application of these approaches remains hampered by limited response rates, significant immune‐related adverse events and acquired resistance mechanisms (Morad et al. 2021; Wang et al. 2022). The efficacy of ICIs is constrained by intrinsic limitations, including patient‐specific checkpoint expression patterns and immunosuppression driven by the tumour microenvironment (TME), which collectively restrict their benefits to only a minority of eligible patients (Zhang and Zhang 2020; Morad et al. 2021; Xiao et al. 2020). Similarly, CAR‐T therapy efficacy in solid tumours is constrained by antigen heterogeneity and immunosuppressive niche formation (Baker et al. 2023; Liu et al. 2022). These challenges underscore an urgent need for innovative strategies that reprogram the tumour‐immune interface to enhance the potency and applicability of anti‐tumour immunity.

Effective anti‐tumour immunity depends fundamentally on the coordinated induction of potent cytotoxic T lymphocyte (CTL) responses and the establishment of a permissive immunostimulatory microenvironment. However, tumours evolve sophisticated adaptive mechanisms, including antigenic drift via neoantigen loss (Al Bakir et al. 2025; Lee et al. 2026), functional subversion of immune cells (Zhang and Zhang 2020; Bader et al. 2020), and epigenetic silencing of programmed cell death pathways (Ibrahim et al. 2019), that collectively disrupt this delicate immunological equilibrium. Although existing immunotherapeutic approaches can enhance T cell priming (Kraehenbuehl et al. 2022; Zhang et al. 2022), they frequently fail to achieve durable control over tumour recurrence and metastasis, largely due to the dual impediments of T cell dysfunction and profoundly microenvironment resistance. Moreover, most current immunotherapeutic agents are designed to target single pathways, rendering them insufficient to comprehensively counteract the multifaceted immune evasion strategies employed by tumours (Kraehenbuehl et al. 2022; Wang et al. 2022). This intricate interplay between immunity and tumour underscores the biological imperative for therapeutic strategies capable of synchronously amplifying cytotoxic T cell responses while reprogramming the immunosuppressive network within an integrated nanoplatform.

Pyroptosis, a gasdermin‐mediated form of immunogenic cell death, has emerged as a promising mechanism for anti‐tumour therapy (Wang et al. 2020). By triggering explosive antigen release and instigating cytokine storm, pyroptosis not only releases concealed tumour antigens but also recruits and activates dendritic cells (DCs), thereby amplifying antigen cross‐presentation and CTL priming (Wei et al. 2023; Zhang et al. 2021). Gasdermin proteins act as molecular executors of pyroptosis, yet tumours frequently suppress their expression as an immune evasion strategy (Loveless et al. 2021; Ma et al. 2018; Wang et al. 2018). Although epigenetic modulators like decitabine can restore gasdermin expression, their off‐target effects often result in severe systemic toxicity (Ding et al. 2024; Wang et al. 2017). Similarly, bacterial lipopolysaccharide (LPS), a potent immune adjuvant, can trigger pyroptosis via the non‐canonical inflammasome pathway upon cytosolic access (Kumari et al. 2023). Nevertheless, current pyroptosis‐focused approaches largely overlook concomitant immunosuppressive signals within the TME, thereby failing to effectively convert antigen release into sustained CTL‐mediated anti‐tumour immunity (Kraehenbuehl et al. 2022; Zhang et al. 2022).

**SCHEME 1:**
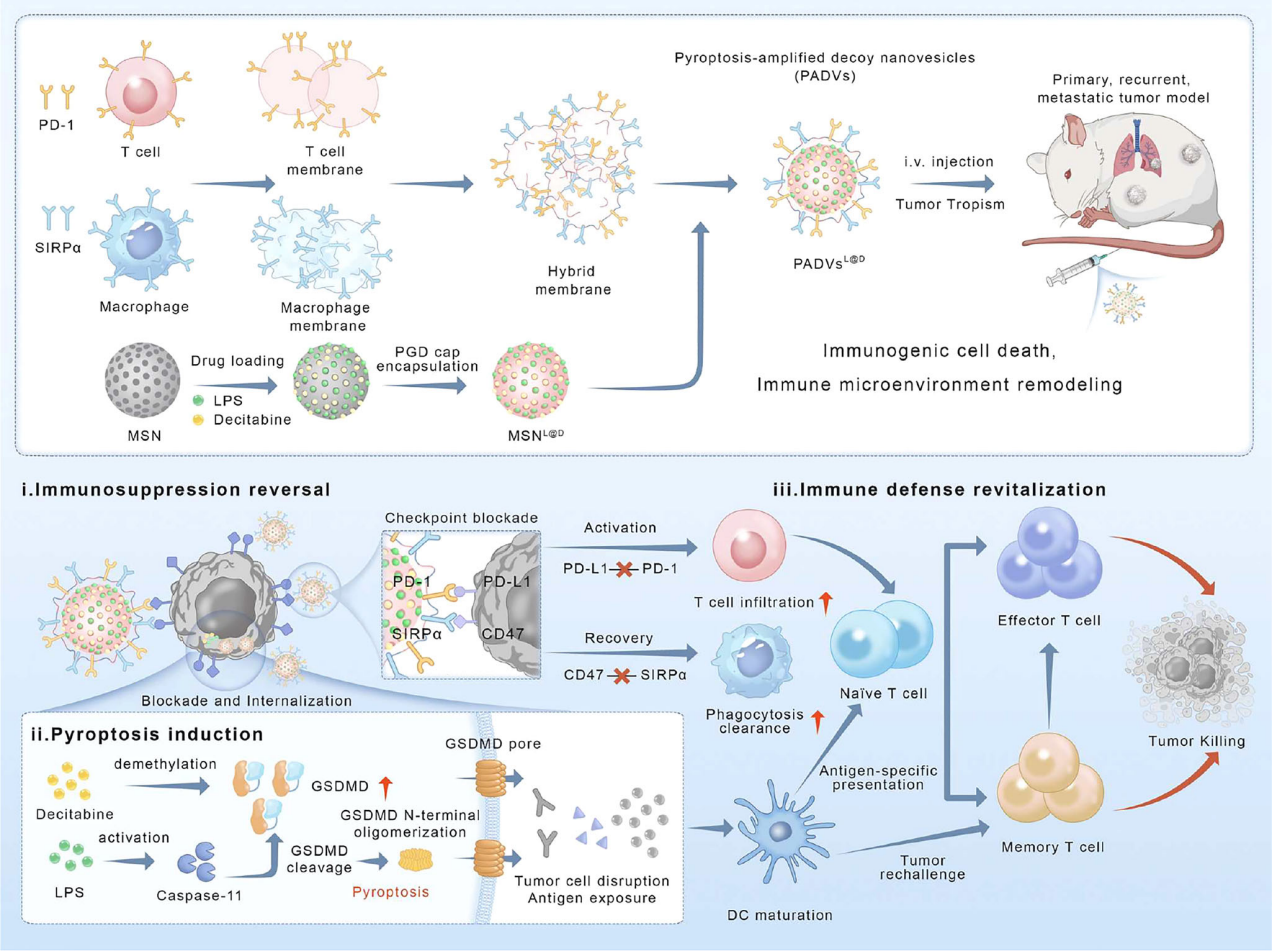
Mechanism of pyroptosis‐amplified decoy nanovesicles (PADVs) for revitalizing anti‐tumour immunity. PADVs reprogram the tumour immune microenvironment and establish endogenous antigen‐specific anti‐tumour immune responses in primary, recurrent, and metastatic tumour models through checkpoint blockade and potent pyroptosis induction.

To address these challenges, we engineered pyroptosis‐amplified decoy nanovesicles (PADVs), a biomimetic platform designed to synchronously reignite pyroptotic cell death and remodel the immunosuppressive microenvironment. PADVs feature a modular architecture consisting of a chimeric decoy membrane shell for neutralizing checkpoint‐mediated immunosuppression, and a nanoparticle core for spatially controlled initiation of pyroptosis. Upon systemic administration, PADVs preferentially penetrate and accumulate in tumours and secondary lymphoid organs, hijacking PD‐1/PD‐L1 and CD47/SIRPα checkpoints to reinvigorate exhausted T cells while boosting further immune activation via costimulatory signalling. Concurrently, PADVs mediate cytosolic co‐delivery of decitabine and LPS, enabling epigenetic potentiation of gasdermin D (GSDMD) and caspase‐11‐driven cleavage to ignite tumour‐intrinsic pyroptosis. This coordinated strategy transforms tumour immune resistance mechanism into immune‐activating signals, synergistically generating tumour‐specific antigen storms that drive DC cross‐presentation and CTLs expansion. In preclinical models, PADVs efficiently suppressed tumour growth, recurrence and metastasis, which elicited a robust immune response to induce short‐term tumour regression and establish long‐term immune memory (Scheme 1). This platform embodies a ‘defense conversion’ strategy that repurposes tumour evasion mechanisms into therapeutic decoys, thereby reawakening host immunity and establishing endogenous antigen‐specific defense through biomimetic interception and precision delivery. This study provides a blueprint for next‐generation immunotherapies that synergistically reprogram immunosuppressive microenvironments and amplify immunogenic cell death, offering a paradigm‐shifting approach to overcome the stubborn immune tolerance in solid tumours.

## 2 Results

### 2.1 GSDMD Potentiates T Cell Immunity and Predicts Melanoma Prognosis

**FIGURE 1:**
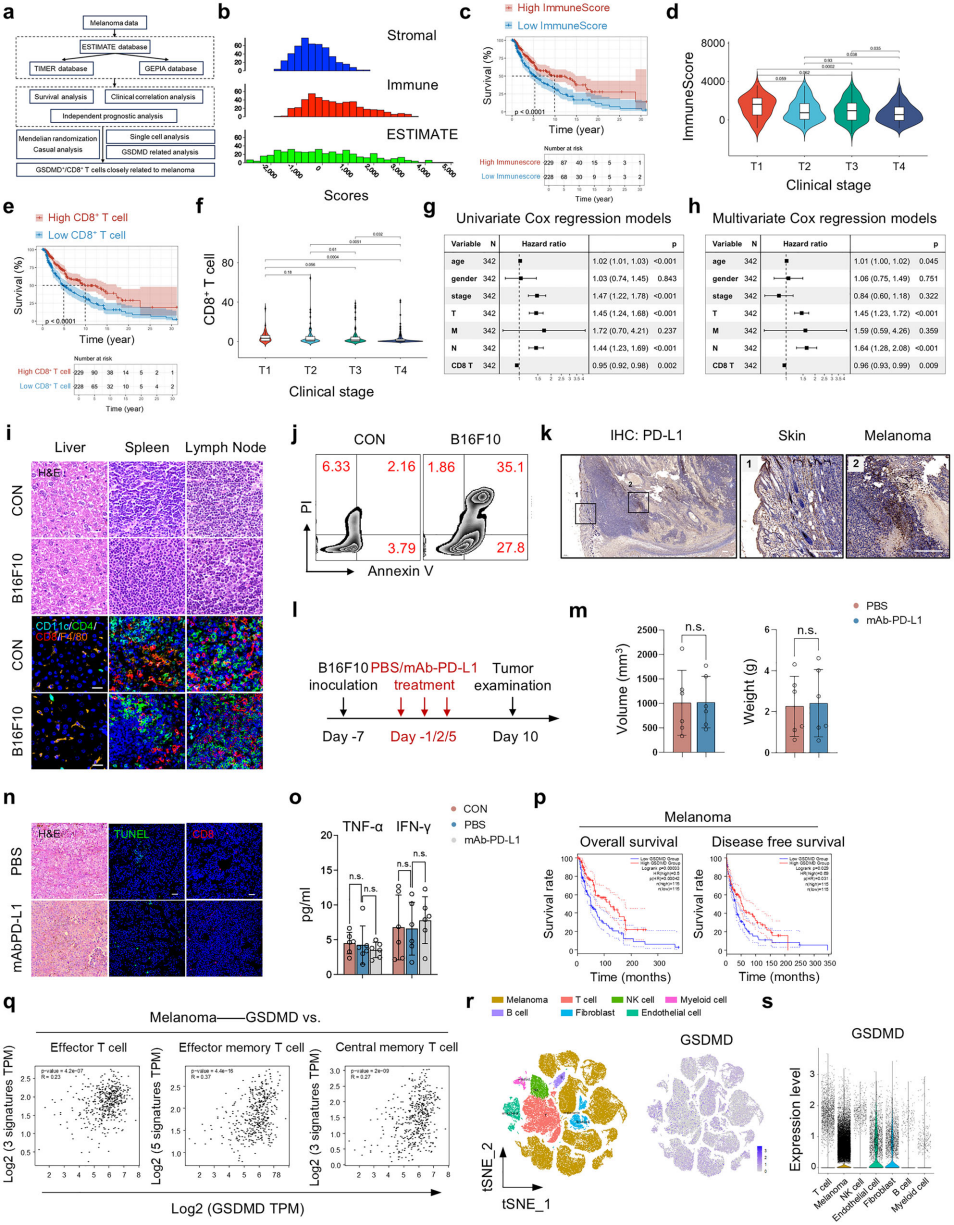
Prognostic relevance of CD8^+^ T cells and GSDMD in melanoma by bioinformatics analysis. (a) Analytical workflow for assessing prognostic relevance of CD8^+^ T cells and GSDMD in melanoma. (b) Tumour purity estimation via the ESTIMATE melanoma database. (c) Kaplan–Meier analysis of the association between ImmuneScore and melanoma prognosis. (d) Correlation analysis of the relationship between ImmuneScore and clinical outcomes. (e) Kaplan–Meier curves demonstrating the independent prognostic value of CD8^+^ T cells infiltration in melanoma, with fractions quantified using the MCPcounter algorithm in the TIMER database. (f) Correlative analyses of the clinical relevance of CD8^+^ T cells fraction. (g, h) Univariate (g) and multivariate (h) Cox regression models assessing the independent predictive power of CD8^+^ T cells for melanoma prognosis. (i) H&E and immunofluorescence analysis of the immune infiltrating levels in liver, spleen and lymph nodes. Scale bar, 20 µm. (j) Apoptosis rate of T cells co‐cultured with B16F10 cells analysed by FACS. (k) Immunohistochemistry analysis of PD‐L1 level in skin and melanoma. Scale bar, 200 µm. (l) Experimental scheme of melanoma‐bearing mice treated with anti‐PD‐L1 (aPD‐L1). (m) Tumour volume and weight at D10 (*n* = 6). (n) H&E, TUNEL and CD8 staining of tumour. Scale bar, 50 µm. (o) TNF‐α and IFN‐γ levels in tumours (*n* = 6). (p) Kaplan–Meier curves demonstrating the independent prognostic value of GSDMD on overall survival (OS) and disease‐free survival (DFS) in GEPIA‐derived melanoma patients. (q) Pearson correlation analysis of the relationship between GSDMD expression and the signatures of effector T cells, effector memory T cells, and central memory T cells in melanoma. (r, s) Single‐cell analysis exhibiting the cellular distribution of GSDMD expression in primary melanoma tissues. n.s., not significant. Exact *p* values were calculated using unpaired two‐tailed Student's *t*‐test (m) and one‐way ANOVA with Tukey's post hoc test (o).

Melanoma is an aggressive malignancy that evades immune surveillance through multiple mechanisms, leading to high mortality rates (Mao et al. 2021). To dissect the immune landscape of melanoma, we employed integrated bioinformatics approaches to systematically decipher the relationship between TME immune components and clinical prognosis (Figure 1a). Analysis of the ESTIMATE melanoma database revealed a positive correlation between elevated ImmuneScore and improved patient survival (Figure 1b,c), whereas declining ImmuneScore coincided with gradual deterioration in histological T‐stage (Figure 1d). Notably, CD8^+^ T cells and CTLs were identified as key predictors of favourable prognosis and reduced disease progression (Figure 1e,f; Figure S1a,b). Univariate and multivariate Cox regression models confirmed CD8^+^ T cells and CTLs as independent protective factors in the melanoma cohort (*p* < 0.05 and Hazard ratio < 1; Figure 1g,h; Figure S1c,d). To delve deeper into the causal relationship between T cell subsets and melanoma risk, Mendelian randomization analysis was performed to identify specific CD8^+^ T cell subsets with protective causal effects, including SSC‐A on HLA DR^+^ CD8br, TD CD8br %CD8br, CM CD8br AC, CD8 on CD39^+^ CD8br, HLA DR^+^ CD8br %T cell and HLA‐DR^+^ CD8br AC (*β* < 0, OR < 1, *p* < 0.05; Figure S1e). Among these, TD CD8br %CD8br (tumour‐responsive CD8^+^ T cells) and HLA‐DR^+^ CD8br AC (activated CD8^+^ T cells) showed consistent associations across independent datasets (Figure S1f–k), reinforcing a causal protective role of functional T cells in melanoma progression and clinical outcomes. This convergent evidence from independent scRNA‐seq datasets across diverse malignancies reinforces the biological plausibility of these T‐cell subset associations and bolsters their translational relevance to melanoma prognosis and therapeutic targeting (Reina‐Campos et al. 2023; Sun et al. 2022; Tagore et al. 2025).

We further interrogated the tumour‐immune interplay using melanoma‐bearing mouse models. Strikingly, tumour progression exerted minimal impact on systemic immunity, with no significant immune activation observed in major lymphoid organs (liver, spleen and lymph nodes) or within tumour tissues (Figure 1i; Figure S1l,m). In vitro co‐incubation assay validated that B16F10 melanoma cells promoted T cell apoptosis to establish immune tolerance (Figure 1j), a mechanism associated with high programmed cell death 1 ligand 1 (PD‐L1) expression on melanoma cells (Figure 1k; Figure S1n). However, PD‐L1 monotherapy failed to elicit tumour regression or immune activation in vivo (Figure 1l–o), suggesting that checkpoint blockade alone is insufficient in immune‐resistant melanoma.

Beyond the establishment of effective immune activation, effective anti‐tumour responses additionally require adequate recognition of tumour‐specific antigens, a process severely hindered by extensive tumour mutational diversity. GSDMD‐mediated pyroptosis circumvents this limitation by forcibly exposing endogenous tumour antigens through its pore‐forming activity, thus holding significant promise for tumour therapy. In melanoma patients, elevated GSDMD expression strongly correlated with prolonged overall survival (OS) and disease‐free survival (DFS) (Figure 1p). Furthermore, we also observed a strong positive correlation between GSDMD expression and the enrichment of gene signatures specific to effector T cells, effector memory T cells and central memory T cells within the melanoma TME (Figure 1q). Notably, this association has not been previously documented, suggesting that GSDMD may play a previously unrecognized role in regulating T cell memory differentiation and effector functional plasticity in the TME. Single‐cell transcriptomics revealed widespread GSDMD expression across multiple cell types, with particularly low basal levels observed in melanoma cells (Figure 1r,s). Functional enrichment analysis implicated GSDMD in cytokine signalling, inflammasome activation, and NF‐κB pathways (Figure S1o), and single‐cell interactome profiling highlighted GSDMD^+^ melanoma cells as active participants in immune‐stimulatory crosstalk with CD8^+^ T cells (Figure S1p). These trends were consistently recapitulated across multiple tumour types (Figure S2a–f). Detailed immune subset quantification further revealed that effector T cell frequencies in these tumours were comparable to those in adjacent normal tissues, whereas naive T cell populations were moderately elevated (Figure S2g,h), underscoring the need for strategies to enhance quiescent naive T cells activation. Further analysis confirmed significantly reduced GSDMD expression in malignant melanoma tissues compared to normal tissues (Figure S2i). Evidence indicates that methylation serves as a prevalent mechanism for GSDMD suppression in tumours (Wang et al. 2018). Notably, GSDMD methylation levels showed progressive elevation with advancing disease stage (from N1 to N3) (Figure S2j). Moreover, aged tumour‐bearing patients frequently exhibited high methylation level coupled with low GSDMD expression (Figure S2k,l), a molecular signature strongly associated with poor prognosis. Specifically, GSDMD expression is epigenetically silenced in many tumours, which could be restored by decitabine (Dec)‐mediated demethylation (Figure S2m,n; Wang et al. 2017). However, the clinical application of decitabine is hampered by severe hematologic toxicity (Nie et al. 2024). While previous studies have established the conceptual framework of pyroptosis‐induced antitumour immunity (Wang et al. 2020), significant challenges remain in translating this strategy into clinical practice due to tumour type‐ and patient‐specific heterogeneity in both GSDMD levels and immunosuppressive TME landscapes. Given the prognostic significance of GSDMD in antitumour immunity, there is an urgent need to develop a tunable and integrated therapeutic platform capable of simultaneously restoring the pyroptotic machinery and reversing the immunosuppressive TME in a context‐dependent manner.

### 2.2 Biomimetic Design and Characterization of Pyroptosis‐Amplified Decoy Nanovesicles

**FIGURE 2:**
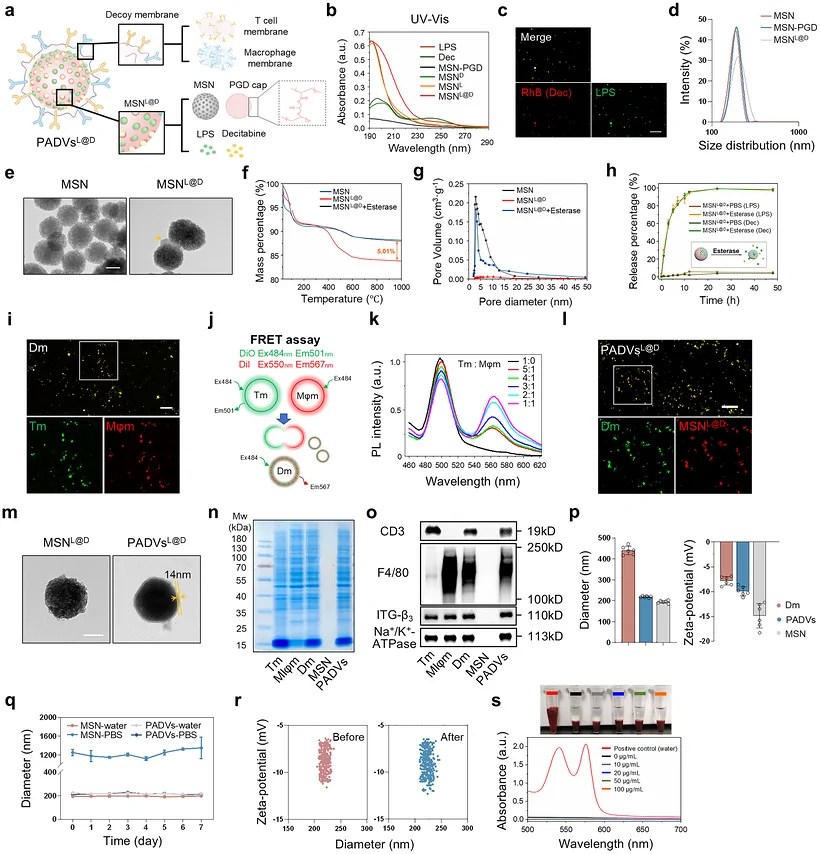
Fabrication and performance of pyroptosis‐amplified decoy nanovesicles (PADVs). (a) Schematic diagram shows the preparation process of PADVs. (b) UV‐Vis spectrometry of LPS, Dec, MSN‐PGD, MSN^L^, MSN^D^ and MSN^L@D^. MSN‐PGD: MSN with PGD cap/no drug; MSN^L^: MSN with LPS only; MSN^D^: MSN with Dec only; MSN^L@D^: MSN with LPS/Dec. (c) The fluorescence co‐location of LPS and RhB (Dec mimics) in MSN. Scale bar, 5 µm. d‐e DLS analysis (d) and TEM image (e) of MSN and MSN^L@D^. The yellow arrow marks the PGD cap. Scale bar, 100 nm. (f, g) TGA (f) and pore size distribution (g) of MSN and MSN^L@D^ before and after treatment by esterase. (h) The release profile of LPS and Dec from MSN^L@D^ with or without esterase. (i) The fluorescence co‐location of T cell membrane and macrophage membrane. Scale bar, 10 µm. (j, k) FRET assay showed the membrane fusion degree. (l) The fluorescence co‐location of decoy membrane and MSN^L@D^. Scale bar, 10 µm. (m) TEM image of MSN^L@D^ and PADVs^L@D^. The yellow arrow marks the decoy membrane. Scale bar, 100 nm. (n, o) The membrane protein analysis by Coomassie blue staining (n) and Western blotting (o). (p) Size distribution and zeta‐potential of Dm, MSN and PADVs detected by DLS (*n* = 6). (q) Stability analysis of MSN and PADVs in water and PBS for 7 days (*n* = 6). (r) Long‐term stability analysis of PADVs after lyophilization. (s) Hemocompatibility analysis of PADVs by the hemolysis test.

To leverage the conserved anti‐tumour role of GSDMD‐mediated pyroptosis, we engineered PADVs as an integrated platform to synergistically reverse tumour immune evasion and trigger immunogenic cell death. PADVs adopt a modular ‘core‐shell’ architecture (Figure 2a), comprising: (1) a decoy membrane shell derived from functionally activated T cells and macrophages, conferring intrinsic tumour‐targeting capacity, immunomodulatory properties, and checkpoint blockade functions; and (2) a tunable mesoporous silica nanoparticle (MSN) core serving as a stimuli‐responsive drug depot for spatiotemporally controlled delivery of pyroptosis inducers.

MSNs were synthesized and loaded with LPS and Dec via physical deposition, followed by capping with a polymerized glyceryl dimethacrylate (PGD) layer (Figure 2a). Successful loading was confirmed by characteristic UV‐Vis peaks at 192 nm (LPS) and 245 nm (Dec) (Figure 2b), along with x‐ray photoelectron spectroscopy (XPS) signals for P 2p (from LPS) and C 1s (C═N)/N 1s (pyridinic nitrogen) (from Dec) (Figure S3a). Fluorescence labelling visually validated the effective drug encapsulation (Figure 2c), with a dual‐drug encapsulation efficiency of 50% achieved at an optimal LPS/Dec/MSN mass ratio of 0.05:0.06:1 (Figure S3b,c). The spherical morphology and uniform size (≈200 nm) were preserved post‐loading, as shown by dynamic light scattering (DLS) and transmission electron microscopy (TEM) (Figure 2d,e). For spatiotemporal drug release control, mesopores were sealed with polyester‐based PGD (Figure 2f,g), retaining >90% of payloads under physiological conditions (Figure S3d). In contrast, esterase exposure triggered PGD degradation and pore reopening (Figure 2f,g), enabling controlled release kinetics (Figure 2h).

For membrane preparation, immunocompetent T cell membrane (Tm) and macrophage membrane (Mφm) were isolated from IFN‐γ/LPS‐stimulated cells to enrich PD‐1 and SIRPα expression for trapping tumour‐derived PD‐L1 and CD47 (Figure S3e–n). These membranes were fused into hybrid decoy membrane (Dm) via co‐extrusion (Figure S3o), which was verified via fluorescence colocalization and FRET assay (Figure 2i–k; Figure S3p). The Dm was subsequently decorated onto MSN^L@D^ at an optimal 2:1 mass ratio (membrane: MSN; Figure S3q), achieving 96% coating efficiency (Figure S3r). Core‐shell structure and membrane protein retention of PADVs were confirmed by fluorescence microscopy, TEM, and Western blot (Figure 2l–o; Figure S3s), with purity confirmed by the absence of intracellular proteins and organelle‐associated proteins (Figure S3t). Dm camouflage increased hydrodynamic size by 24 nm and shifted zeta potential from −14.85 ± 2.45 mV (MSN) to −9.9 ± 0.94 mV (Figure 2p; Figure S3u). This biomimetic modification significantly improved colloidal stability across diverse media, minimizing aggregation and deposition (Figure 2q), and conferred freeze‐drying tolerance for convenient long‐term storage (Figure 2r). Additionally, PADVs exhibited high hemocompatibility, serum stability, and negligible cytotoxicity up to 100 µg/mL (Figure 2s; Figure S3v,w).

Collectively, these results demonstrate the successful fabrication of PADVs as a stable, biocompatible, and multifunctional nanoplatform for coordinated pyroptosis induction and immune reprogramming.

### 2.3 PADVs Counteract Tumour Immune Evasion Through Coordinated Checkpoint Blockade and Pyroptosis Rewiring

**FIGURE 3:**
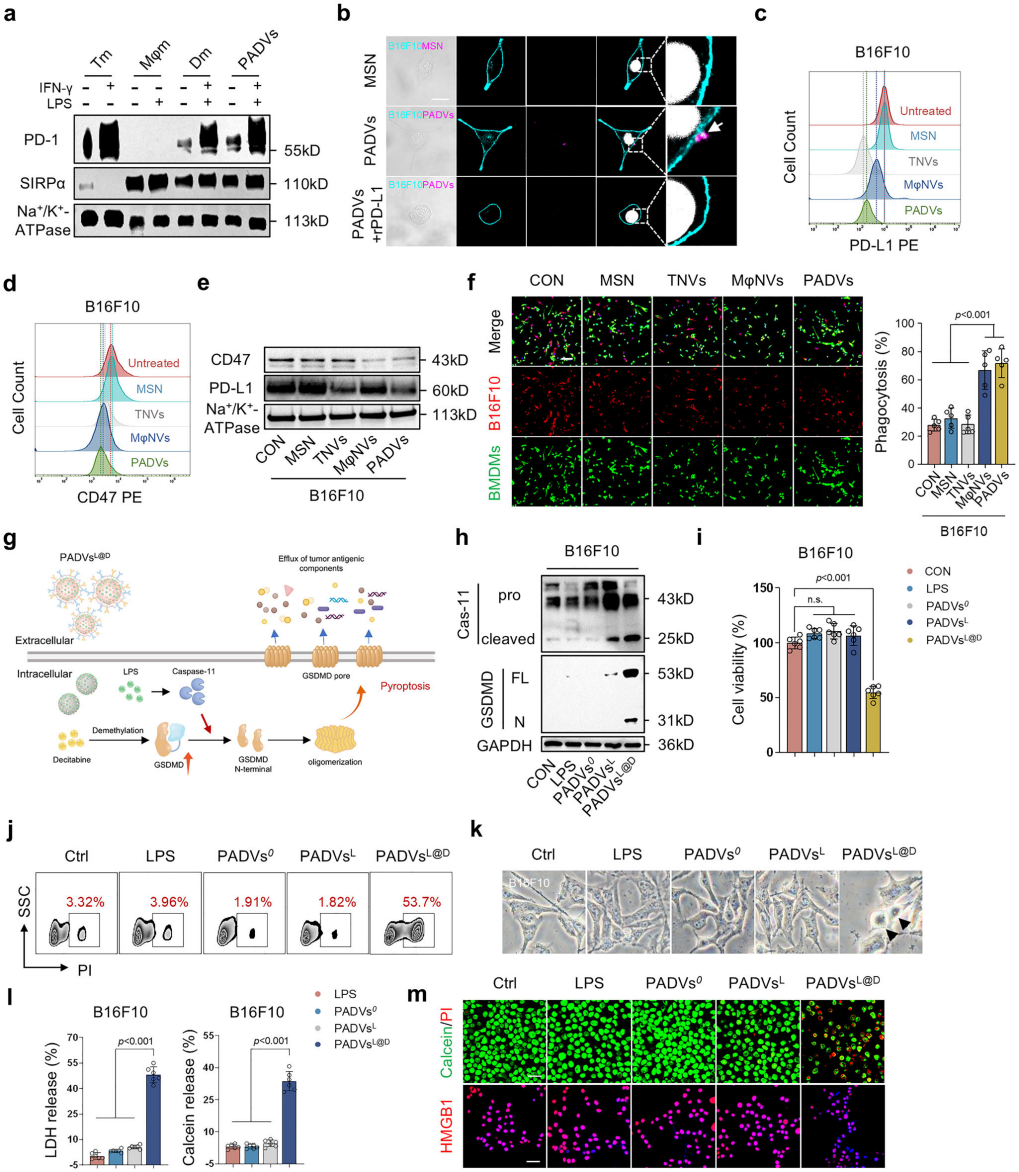
PADVs^L@D^ eliminate tumour cells in vitro through dual checkpoint blockade and pyroptosis induction. (a) Western blotting analysis of PD‐1 and SIRPα expression on PADVs. (b) Fluorescence visualization of PADVs‐B16F10 cell interaction. Scale bar, 20 µm. (c, d) Flow cytometric quantification of PD‐L1 (c) and CD47 (d) expression on B16F10 cells post‐PADVs treatment. (e) Western blotting analysis of PD‐L1 and CD47 levels on tumour cell surface. (f) Detection of macrophage phagocytosis efficiency for tumour cells treated with different formulations (*n* = 5). Scale bar, 100 µm. (g) Schematic diagram of the mechanism that PADVs^L@D^ induced pyroptosis of tumour cells. (h) Western blotting analysis of caspase‐11 activation and GSDMD cleavage in B16F10 cells after different treatments. (i) Cell viability test of B16F10 cells after different treatments (*n* = 6). (j) PI‐positive rate of B16F10 cells was analysed by flow cytometry. (k) Representative photos of B16F10 cells after different treatments. Black arrow indicates typical pyroptotic cells. (l) LDH and calcein release assay of B16F10 cells after different treatments (*n* = 6). (m) Live/dead cell staining (Calcein AM/PI) and HMGB1 translocation imaging of B16F10 cells after different treatments. Scale bar, 50 µm. n.s., not significant. Exact *p* values were calculated using one‐way ANOVA with Tukey's post hoc test (f, i) or Dunnett T3 post hoc test (l).

Surface protein analysis confirmed that PADVs inherited high‐density PD‐1 and SIRPα from activated T cells and macrophages (Figure 3a). PD‐1/PD‐L1 axis‐mediated specific binding to B16F10 cells was confirmed through competitive inhibition with recombinant PD‐L1 (Figure 3b; Figure S4a). PADVs effectively downregulated surface PD‐L1 and CD47 on tumour cells through competitive receptor engagement (Figure 3c–e). Attenuation of CD47 signalling enhanced macrophage phagocytosis of tumour cells by 2.59‐fold compared to untreated controls (Figure 3f). PADVs^L@D^ further mediated cytosolic co‐delivery of LPS and Dec into tumour cells, initiating a pyroptotic cascade through Dec‐mediated GSDMD demethylation and LPS‐triggered caspase‐11 activation (Figure 3g; Rathinam et al. 2019; Shi et al. 2014). Notably, free LPS, free decitabine, or their combination failed to induce pyroptosis, as the pyroptosis‐inducing activity requires cytosolic delivery of LPS and decitabine (Figure S4b–d). In contrast, PADVs^L@D^ ensured GSDMD upregulation and N‐terminal fragment accumulation by Western blotting (Figure 3h). Furthermore, while empty PADVs (PADVs*^0^*) or LPS‐loaded PADVs (PADVs^L^) alone showed negligible cytotoxic effects, PADVs^L@D^ induced over 50% cytotoxicity via GSDMD‐dependent pyroptosis (Figure 3i,j). Distinctive balloon‐like swelling morphology was observed exclusively in PADVs^L@D^ group (Figure 3k), accompanied by measurable LDH/calcein release and HMGB1 nuclear‐to‐cytoplasmic translocation (Figure 3l,m; Figure S6a). This multimodal evidence confirms pyroptosis execution through caspase‐11/GSDMD axis activation by PADVs^L@D^.

We further validated the broad applicability of PADVs in 4T1 (breast cancer) and CT26 (colon cancer) cell models, which exhibit distinct basal GSDMD expression (Figure S2m,n). PADVs consistently downregulated PD‐L1 and CD47 levels (Figure S5a–d,f–i), and enhanced macrophage phagocytosis (Figure S5e,j) across both lines. Intriguingly, 4T1 cells with low basal GSDMD required Dec‐mediated epigenetic priming for pyroptosis induction (60.07 ± 5.95% death), whereas CT26 cells with intrinsically high GSDMD expression underwent significant pyroptosis (54.96 ± 21.13%) with PADVs^L^ alone (Figure S6), revealing that cell‐specific GSDMD methylation status dictates therapeutic responsiveness.

Collectively, these findings demonstrate PADVs as a multimodal nanoplatform that synergizes bio‐interceptive checkpoint blockade with epigenetic restoration of pyroptosis, resuscitating potent tumour‐intrinsic pyroptotic cascades across diverse malignancies.

### 2.4 PADVs Directly Activate the Innate and Adaptive Immune Responses In Vitro

Tumours frequently evade immune surveillance by concurrently suppressing innate and adaptive immune responses. Leveraging the hybrid T cell‐macrophage membrane composition, we systematically elucidated the immunomodulatory capacity of PADVs in reversing the suppressive networks.

**FIGURE 4:**
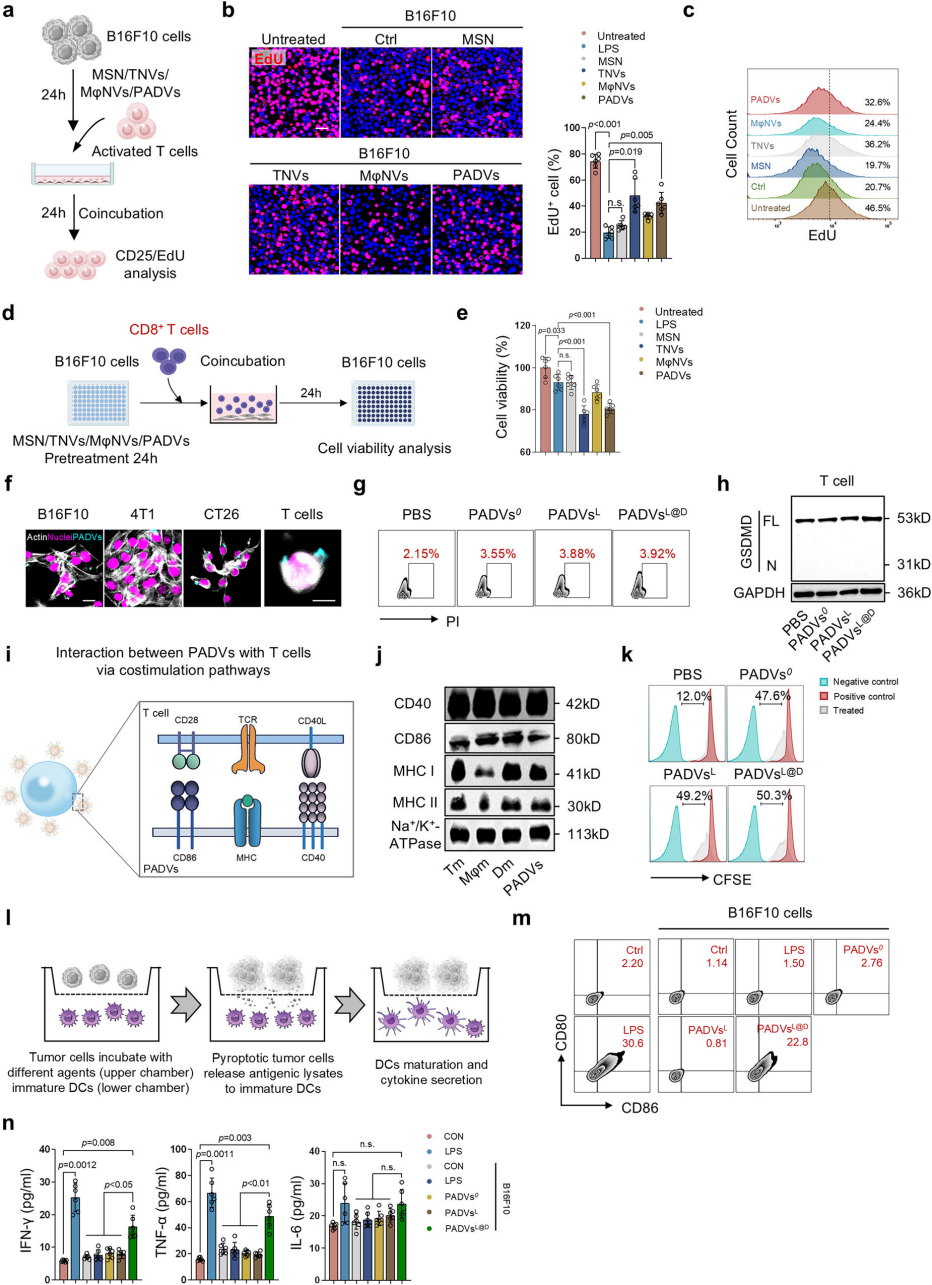
PADVs revitalize anti‐tumour immunity via synergistic amplification of immune exhaustion reversal, antigen‐specific presentation, and immune costimulation. (a) Schematic illustration of the experimental design to evaluate PADVs’ efficacy in rescuing B16F10‐induced T cell anergy. (b, c) EdU incorporation assay showing T cell proliferation after co‐culture with PADVs‐treated B16F10 cells analysed by fluorescence (b) and FACS (c) (*n* = 6). Scale bar, 20 µm. (d, e) Cell viability assay of B16F10 cells with different pretreatments after coincubation with T cells (*n* = 6). (f) Fluorescence images showing the differential interaction pattern of PADVs on T cells versus tumour cells. Scale bar, 20 µm (left) and 5 µm (right). (g) Flow cytometry analysis of PI^+^ population in T cells treated with different PADVs. (h) Western blotting analysis of GSDMD cleavage in T cells after treatment with different PADVs. (i) Diagrams of PADVs‐T cell interactions. (j) The expression of immune costimulatory molecules on PADVs. (k) Quantitative analysis of T cell proliferative responses to different PADVs by CFSE. (l, m) Detection of DC activation status following exposure to pyroptotic B16F10 cells. (n) Detection of cytokines secreted by DCs exposed to pyroptotic B16F10 cells (*n* = 6). n.s., not significant. Exact *p* values were calculated using one‐way ANOVA with Tukey's post hoc test (e) or Dunnett T3 post hoc test (b, n).

To counteract PD‐L1‐driven T cell exhaustion, we evaluated PADVs’ effects on T cell functional recovery (Figure 4a). Owing to their high surface PD‐1 expression, both T cell membrane‐derived nanovesicles (TNVs) and PADVs potently rescued T cell proliferation deficits caused by B16F10 cells, effectively restoring T cell viability and activation, evidenced by elevated EdU incorporation and CD25 expression (Figure 4b,c; Figure S7a). A subsequent cytotoxicity assay confirmed that these rescued T cells regained tumour‐killing capacity, with TNVs and PADVs promoting CD8^+^ T cell‐mediated tumour cell elimination in vitro (Figure 4d,e), validating their ability to disrupt PD‐L1‐mediated immunosuppression and restore T cell efficacy. We further investigated whether PADVs directly exert immunomodulatory effects on T cells. Fluorescence imaging revealed that unlike tumour cells, which efficiently internalize PADVs, T cells only bind to PADVs on their surface without detectable internalization (Figure 4f; Figure S7b), thereby avoiding direct pyroptosis induction in T cells (Figure 4g,h; Figure S7c,d). Importantly, PADVs retained parental cell‐derived co‐stimulatory molecules (CD40, CD86, MHC I/II) on their surface (Figure 4i,j), which potently promoted T cell proliferation (Figure 4k). Using neutralizing antibodies against these co‐stimulatory molecules, we demonstrated that PADVs promote T cell proliferation through co‐stimulatory signalling (Figure S7e), further amplifying the immune response.

As professional antigen‐presenting cells, DCs orchestrate T cell priming through antigen presentation and co‐stimulatory signalling (Wculek et al. 2020). Transwell assays revealed that pyroptotic tumour lysates generated by PADVs^L@D^ treatment significantly boosted activated CD80^+^CD86^+^ DC populations (Figure 4l,m) and stimulated robust secretion of pro‐inflammatory cytokines (Figure 4n).

Collectively, these results demonstrate that PADVs coordinately enhance anti‐tumour immunity by: (1) neutralizing PD‐L1‐mediated T cell exhaustion via competitive checkpoint disruption; (2) amplifying adaptive immune responses via native co‐stimulatory signalling; and (3) promoting DCs activation and maturation via pyroptosis‐derived antigen release.

### 2.5 PADVs Enable Tumour‐Targeted Imaging and Potent Anti‐Tumour Efficacy In Vivo

**FIGURE 5:**
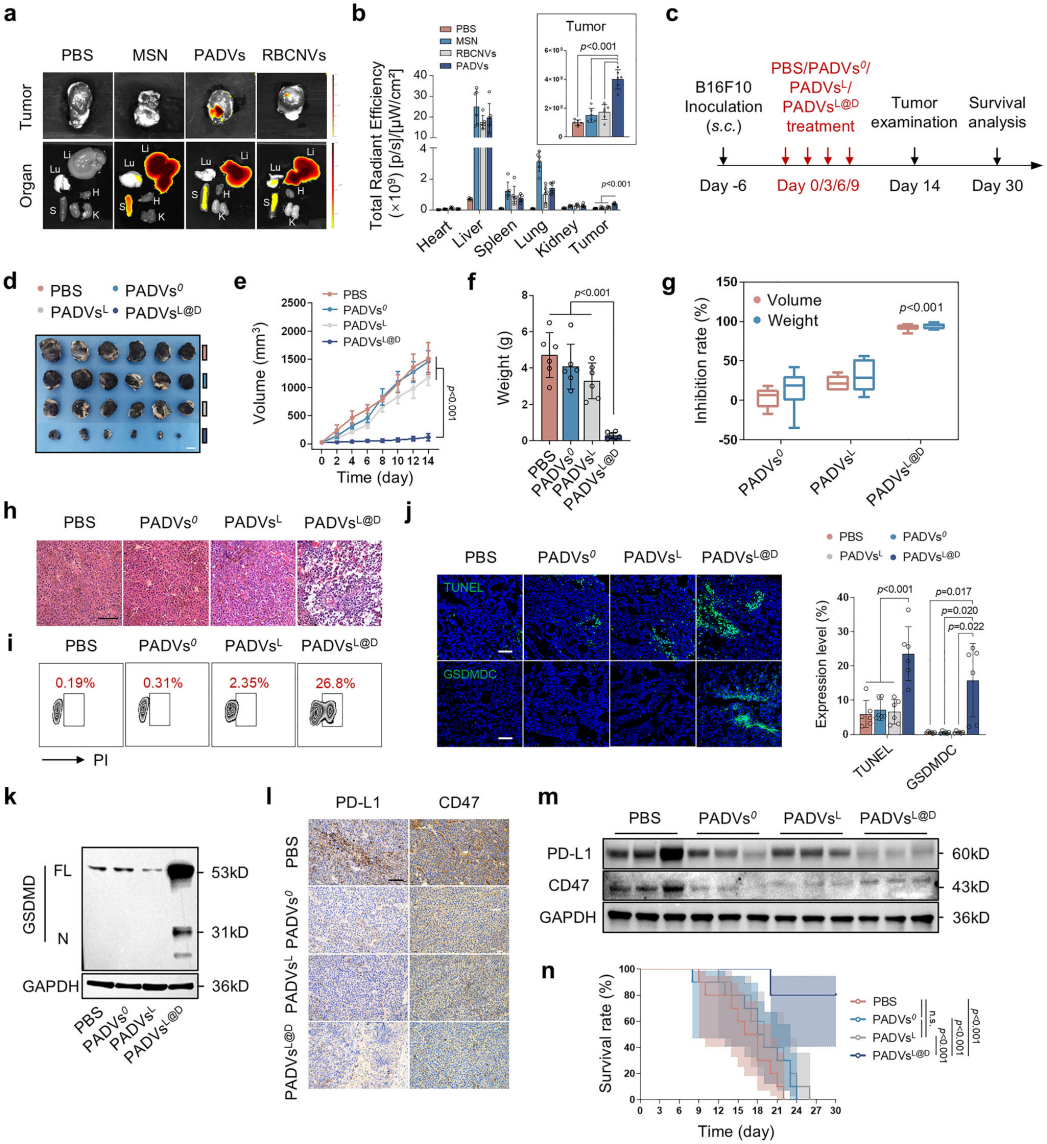
PADVs enable targeted eradication of primary subcutaneous melanoma. (a) The distribution of PADVs in tumour and major organs by in vivo fluorescence imaging. (b) Quantitative analysis of PADVs enrichment levels in tumour and major organs. (c) Schematic of primary subcutaneous melanoma model establishment and therapeutic regimen. (d) Gross morphology of tumours in each group. Scale bar, 1 cm. (e, f) Tumour volume (e) and weight (f) in each group (*n* = 6). (g) Tumour inhibition rate in each group (*n* = 6). (h) Histopathological evaluation of tumour by H&E staining. Scale bar, 100 µm. (i) Flow cytometric quantification of PI^+^ dead cells in tumour tissues. (j) TUNEL and GSDMDC staining of tumours after treatment. Scale bars, 100 µm. (k) Western blotting analysis of GSDMD cleavage in tumour. (l, m) Immunohistochemistry (l) and Western blotting (m) analysis of PD‐L1 and CD47 levels in tumour. Scale bars, 100 µm. (n) Survival analysis of tumour‐bearing mice in each group until Day 30 (*n* = 10). Exact *p* values were calculated using one‐way ANOVA with Tukey's post hoc test (b, f, g, j) or Dunnett T3 post hoc test (e), Mann–Whitney test (j) and Log‐rank test (n).

To evaluate the in vivo therapeutic potential of PADVs, we first characterized their tumour‐targeting capability, a critical determinant of immunotherapeutic efficacy. Using 3D tumour spheroid models, the enhanced intratumoral penetration of PADVs functionalized with decoy biomembrane was confirmed (Figure S8a). In vivo fluorescence imaging revealed distinct biodistribution patterns dictated by membrane properties (Figure 5a,b; Figure S8b–g). Compared to membrane‐free MSNs, which predominantly accumulated in the liver and spleen, PADVs showed relatively improved tumour‐preferential accumulation. RBC‐derived nanovesicles (RBCNVs), despite exhibiting prolonged circulation, failed to achieve notable tumour accumulation due to a lack of surface chemotactic molecules (Figure 5a,b; Figure S8b–f). To further understand the in vivo behaviour of PADVs, we characterized the protein corona formed on PADVs after serum incubation using comparative proteomics (Figure S9). GO and KEGG analyses of the upregulated proteins in FBS‐incubated PADVs versus naked PADVs demonstrated that PADVs rapidly adsorb abundant plasma proteins, most notably albumin, as well as complement components, coagulation factors, lipoproteins, and extracellular matrix proteins, which collectively modulate vesicle circulation stability, immune response and tumour‐targeting efficiency (Liam‐Or et al. 2024; Dietz et al. 2023). These findings indicate that while the immunocompetent membrane of PADVs confers enhanced tumour homing compared to non‐functionalized nanoparticles, further optimization is needed to improve tumour‐to‐liver distribution ratios. Nevertheless, this inherent tumour‐homing property may still support applications such as real‐time tumour imaging and visualization.

We next established subcutaneous melanoma models to assess therapeutic efficacy (Figure 5c). By Day 14, PADVs^L@D^ significantly inhibited tumour growth by 92.33 ± 4.12% in volume and 94.55 ± 3.71% in weight, respectively, compared with the PBS group (Figure 5d–g). Histological analysis revealed extensive necrotic foci and cellular disintegration exclusively in PADVs^L@D^‐treated tumours (Figure 5h). PI and TUNEL staining confirmed approximately 20% cell death in the PADVs^L@D^ group (Figure 5i,j). At the molecular level, PADVs^L@D^ induced GSDMD cleavage and downregulated PD‐L1 and CD47 expression (Figure 5j–m), synergistically extending the median survival of tumour‐bearing mice from 16 days (PBS group) to over 30 days (PADVs^L@D^ group) (Figure 5n).

To validate broad applicability across tumour types, we further evaluated PADVs in 4T1 breast cancer and CT26 colon cancer models. In 4T1 tumours, PADVs^L@D^ induced approximately 15.0% pyroptosis, achieved 95.34 ± 5.27% volume inhibition and 93.91 ± 5.69% weight inhibition (Figure S10a–i). In CT26 tumours, which exhibit high intrinsic GSDMD expression, PADVs^L^ alone suppressed tumour volume and weight by 86.73 ± 12.30% and 84.20 ± 12.86%, respectively (Figure S10k–s). These results demonstrate that PADVs elicit potent primary tumour suppression (≈90%) through a multiplier effect, despite inducing pyroptosis in only a subset of tumour cells (≈20%).

### 2.6 PADVs Revitalize the Host Anti‐Tumour Immune Microenvironment

**FIGURE 6:**
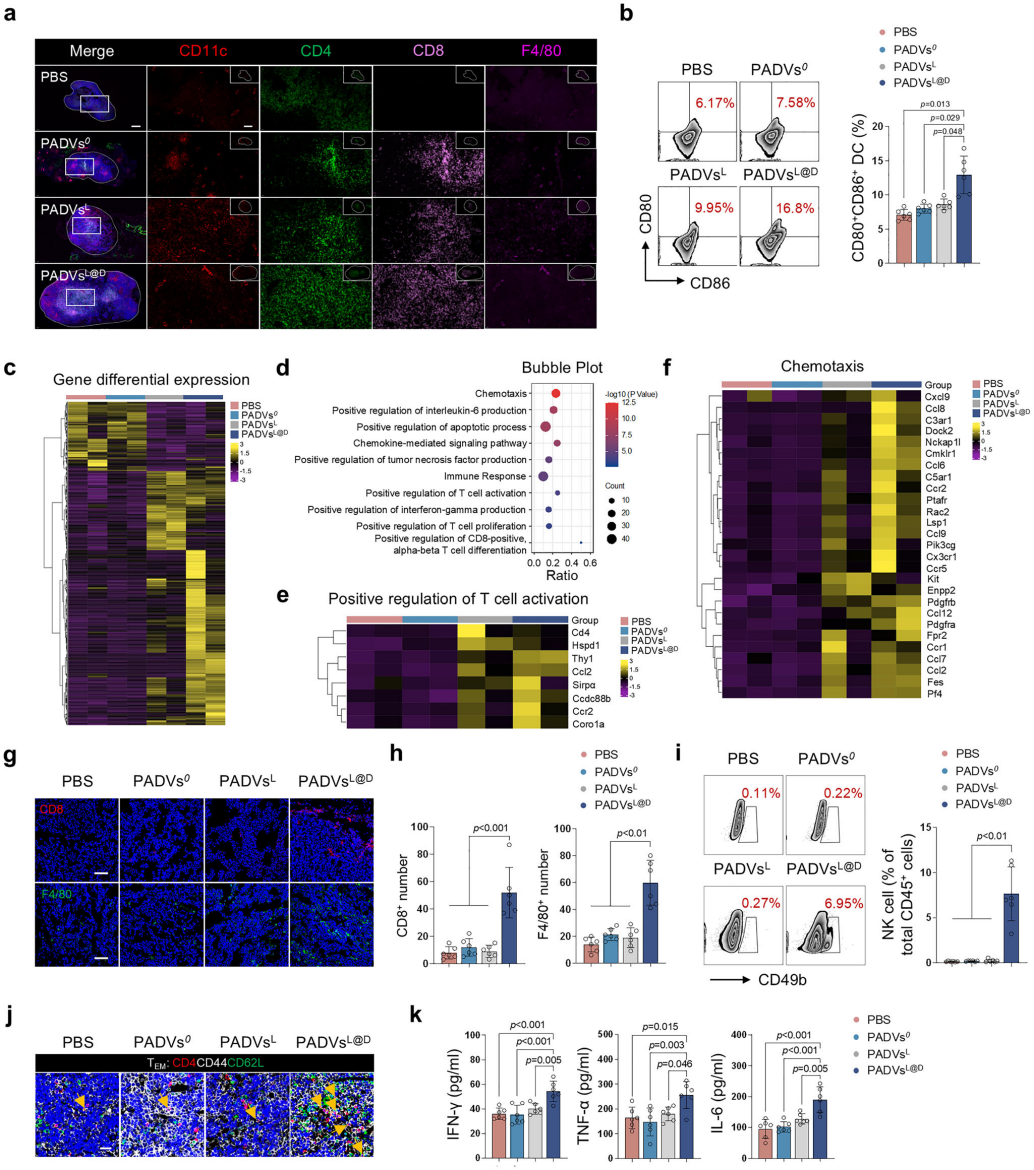
PADVs remodel the anti‐tumour immune microenvironment through systemic immune activation. (a) Multiplex immunofluorescence imaging of immune cell infiltration in draining lymph nodes. Scale bars, 200 µm (left), 50 µm (magnified panel). (b) Quantification of dendritic cell (DC) activation level in lymph nodes (LNs) (*n* = 6). (c) Differential expression landscape highlights the distinct transcriptional signatures elicited by each treatment. (d) Gene ontology enrichment of differentially expressed genes (DEGs) in specific biological pathways. (e, f) Heatmap visualization of DEGs associated with T cell activation (e) and chemotaxis (f) GO term. (g, h) Representative fluorescence images (g) and quantification analysis (h) of intratumoral CD8^+^ T cells and macrophages (*n* = 6). Scale bar, 100 µm. (i) Flow cytometric analysis of intratumoral NK cells level (*n* = 6). (j) Immunofluorescence staining of central memory T cells (CD4^+^CD44^+^CD62L^+^) in the spleen after different treatments. Scale bar, 20 µm. (k) Quantification of intratumoral inflammatory factors (*n* = 6). Exact *p* values were calculated using one‐way ANOVA with Tukey's post hoc test (h, k) or Dunnett T3 post hoc test (b, h, i).

To clarify whether PADVs^L@D^ establishes a durable anti‐tumour microenvironment critical for preventing recurrence and metastasis, we first investigated their effect on lymph node (LN) activation, the key sentinel sites for early immune recognition and priming within tumour‐bearing regions. In vivo imaging revealed PADVs accumulation in tumour‐draining LNs (Figure S11a,b). Multiplex immunofluorescence analysis demonstrated profound LN remodelling following PADVs^L@D^ treatment, characterized by marked immune cell expansion and the formation of abundant CD4^+^/CD8^+^ T cell clusters within paracortical zones (Figure 6a). In addition, PADVs^L@D^ treatment significantly increased the frequency of activated CD80^+^CD86^+^ DCs by 1.83‐fold compared to PBS controls (Figure 6b), with no occurrence of pyroptosis‐related tissue damage (Figure S11c,d).

Transcriptomic profiling of tumour tissues revealed a robust immune remodelling signature in PADVs^L@D^‐treated tumours, with 496 differentially expressed genes (DEGs) identified (Figure 6c). Gene ontology (GO) analysis uncovered profound enrichment in pathways governing chemotaxis and cytokine production (Figure 6d), highlighted by coordinated upregulation of key chemokines (CCL3, CXCL9) and T cell activation markers (CD4) (Figure 6e,f). KEGG analysis further corroborated the activation of TNF signalling and chemokine cascades (Figure S11e), with a core set of chemokine genes, including CCL8, CCL3, CCL2, CCL7 and CXCL9, consistently enriched across these enriched KEGG pathways (Figure S11f,g), pointing to a coordinated chemotactic network orchestrated by PADVs^L@D^. Strikingly, this transcriptional reprogramming translated into profound changes in the tumour immune landscape. Immunofluorescence analysis revealed that PADVs^L@D^ treatment dramatically enhanced intratumoral infiltration of CD8^+^ T cells by 6.64‐fold and macrophages by 4.31‐fold (Figure 6g,h), findings consistently recapitulated in 4T1 and CT26 models (Figure S10j,t). Conversely, immunosuppressive populations, namely regulatory T cells (Tregs) and M2‐polarized macrophages, were significantly reduced (Figure S11h), tipping the immune balance toward an activated state. NK cells were also recruited to the tumour site, further contributing to the anti‐tumour effector response (Figure 6i). Beyond local immune activation, PADVs^L@D^ treatment induced systemic immune memory, as evidenced by the robust formation of central memory T cells (CD4^+^CD44^+^CD62L^+^) in the spleen (Figure 6j). Collectively, these coordinated changes culminated in the reversal of the immunosuppressive TME, marked by elevated intratumoral levels of IFN‐γ, TNF‐α and IL‐6 (Figure 6k).

Together, these findings establish PADVs^L@D^ as a potent orchestrator of immune microenvironment remodelling, capable of not only activating local effector responses but also establishing durable systemic immunity.

### 2.7 PADVs Establish Antigen‐Specific Immune Memory to Inhibit Tumour Recurrence

**FIGURE 7:**
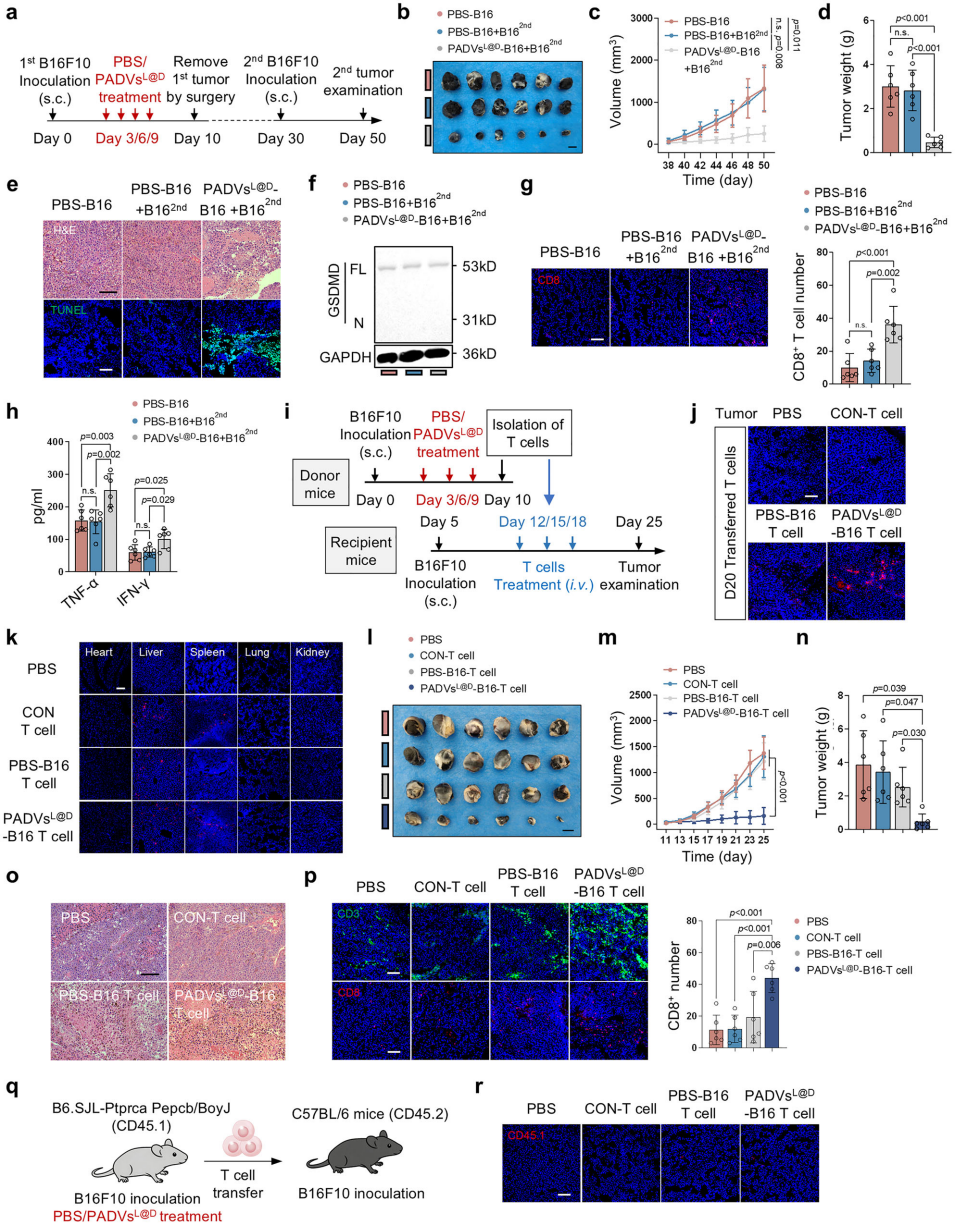
PADVs^L@D^‐induced antigen‐specific immune memory confers resistance to homologous tumour recurrence. (a) Schematic of tumour rechallenge model establishment and therapeutic regimen. (b) Gross morphology of B16F10 tumours in each group. Scale bar, 1 cm. (c, d) Tumour volume (c) and weight (d) in each group (*n* = 6). (e) Histopathological evaluation of tumour tissue by H&E and TUNEL staining. Scale bars, 100 µm. (f) Western blotting analysis of GSDMD cleavage in tumour. (g) Quantification of CD8^+^ T cell infiltration level by immunofluorescence. Scale bar, 100 µm. (h) Quantification of intratumoral inflammatory factors (*n* = 6). (i) Schematic of adoptive T cell transfer experiment verifying anti‐tumour immunity conferred by PADVs^L@D^‐primed T cells. (j, k) Fluorescence images showed the distribution of transferred T cells in tumours (j) and major organs (k). Scale bar, 100 µm. (l) Gross morphology of B16F10 tumours in each group. Scale bar, 1 cm. (m, n) Tumour volume (m) and weight (n) in each group (*n* = 6). (o) Histopathological evaluation of tumour tissue by H&E staining. Scale bar, 100 µm. (p) Fluorescence images showed CD3^+^ and CD8^+^ T cell infiltration levels in the tumour. Scale bar, 100 µm. (q) Schematic of adoptive transfer experiment using CD45.1/CD45.2 congenic mice. (r) Immunofluorescence staining of CD45.1^+^ donor‐derived cells in recipient tumour tissues. Scale bar, 100 µm. n.s., not significant. Exact *p* values were calculated using one‐way ANOVA with Tukey's post hoc test (d, g, h, m, p) or Dunnett T3 post hoc test (c, n).

To substantiate the establishment of durable anti‐tumour immunity by PADVs^L@D^, the host's resistance ability to tumour recurrence was observed in a tumour rechallenge model. Mice bearing the 1st tumour burden received designated treatments followed by surgical resection of the tumour at Day 10. After a 20‐day recovery period, mice were rechallenged with homologous tumour cells, and tumour growth was monitored for an additional 20 days (Figure 7a). PADVs^L@D^‐pretreated mice exhibited 75.95 ± 22.49% inhibition of 2nd tumour growth compared to PBS controls (Figure 7b–d). In contrast, PBS‐pretreated mice displayed no protective immunity against rechallenge. Histological analysis revealed TUNEL^+^ necrotic foci only in the 2nd tumour from PADVs^L@D^‐pretreated mice (Figure 7e). Notably, no GSDMD cleavage was detected in the 2nd tumours (Figure 7f), confirming recurrence suppression was immunity‐mediated rather than pyroptosis‐dependent. A substantial infiltration of CD8^+^ T cells and elevated intratumoral levels of IFN‐γ and TNF‐α in the 2nd tumours corroborated the activation of robust adaptive immunity during rechallenge (Figure 7g,h). This immune protection was long‐lasting, remaining effective after a 3‐week interval, and strictly antigen‐specific, as evidenced by the absence of tumour inhibition or immune activation when rechallenged with heterologous MC38 tumours (Figure S12).

To further validate the role of immune memory in this protection, an adoptive T cell transfer experiment was designed (Figure 7i). Briefly, donor mice were subcutaneously inoculated with B16F10 tumour cells at Day 0 and received three treatments at Days 3, 6 and 9. At Day 10, splenic T cells were isolated from these donor mice. Separately, recipient mice were inoculated with B16F10 tumour cells at Day 5 and received adoptive transfers of T cells from different donor groups at Days 12, 15 and 18. Tumour growth and immune activation were assessed at Day 25. The transferred T cells from PADVs^L@D^‐treated donors exhibited enhanced tumour homing capacity (Figure 7j,k) and demonstrated a potent tumour suppression effect of 89.50 ± 9.68% (Figure 7l–o). Notably, adoptively transferred T cells significantly enhanced T cell infiltration within the TME (Figure 7p). Using the CD45.1/CD45.2 congenic marker system to distinguish donor‐derived from endogenous T cells, we further confirmed that this enhanced infiltration was dominantly attributed to the activation of endogenous anti‐tumour immunity in recipient mice (Figure 7q,r).

Altogether, these results demonstrate that PADVs^L@D^ establishes functional, antigen‐specific immune memory, enabling durable T cell‐mediated protection against tumour recurrence.

### 2.8 PADVs Suppress Hematogenous Metastatic Progression

**FIGURE 8:**
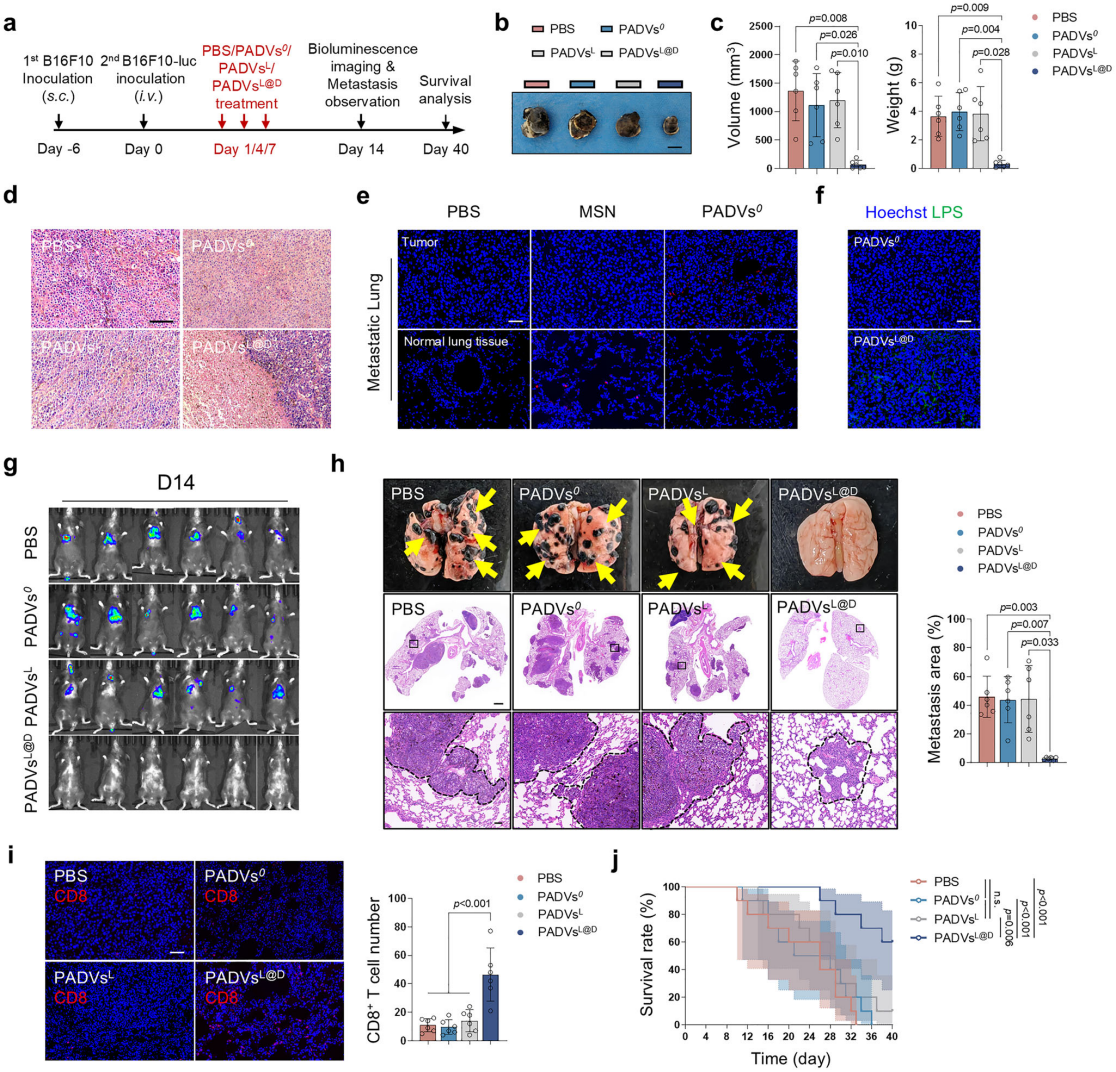
PADVs^L@D^ mitigate hematogenous tumour metastasis and suppress lung metastatic colonization. (a) Experimental schema of B16F10 melanoma hematogenous metastasis model establishment and therapeutic regimen. (b) Gross morphology of primary subcutaneous tumours in each group. Scale bar, 1 cm. (c) The volume and weight of primary tumour at Day 14 in each group (*n* = 6). (d) Histopathological evaluation of primary tumours by H&E staining. Scale bar, 100 µm. (e) Fluorescence tracking of PADVs distribution in metastatic lesions versus normal lung tissue. Scale bar, 100 µm. (f) Fluorescence images showing LPS delivery to metastatic lesions by PADVs. Scale bar, 100 µm. (g) Bioluminescence imaging of lung metastatic burden using B16F10‐luc cells at Day 14 (*n* = 6). (h) Gross morphology of lung with metastatic nodules (yellow arrows), and whole‐lung H&E staining with micrometastatic histology (dotted line). Scale bars, 1000 µm (middle), 50 µm (bottom). (i) Quantification of CD8^+^ T cell infiltration level in metastatic tumour foci. Scale bar, 100 µm. (j) Survival curves of tumour‐bearing mice with hematogenous metastasis in each group until Day 40 (*n* = 10). Exact *p* values were calculated using one‐way ANOVA with Tukey's post hoc test (i) or Dunnett T3 post hoc test (c, h), and Log‐rank test (j).

Given that approximately 50% of mucosal melanoma patients present with metastatic stages (Mao et al. 2021), we evaluated the anti‐metastatic efficacy of PADVs using a hematogenous dissemination model (Figure 8a). In mice bearing both primary tumours and lung metastases, PADVs^L@D^ treatment simultaneously suppressed primary tumour growth (Figure 8b–d), and selectively targeted scattered lung metastases, demonstrating preferential accumulation in metastatic foci (Figure 8e,f). By Day 14, in vivo imaging and gross examination confirmed a significant reduction in metastatic lung nodules following PADVs^L@D^ treatment (Figure 8g,h). Histopathological analysis revealed only minimal residual lesions in PADVs^L@D^‐treated mice versus diffuse infiltration in controls (Figure 8h). This anti‐metastatic effect correlated with a 4.21‐fold increase in CD8^+^ T cell infiltration within lungs (Figure 8i), extending median survival from 26 days (PBS group) to over 40 days (PADVs^L@D^ group) (Figure 8j).

We further validated the broad‐spectrum anti‐metastatic efficacy of PADVs in CT26 colon cancer models. PADVs^L^ similarly inhibited subcutaneous tumour growth and pulmonary metastasis (Figure S13a–h), activated the CD8^+^ T cell response and significantly improved survival outcomes (Figure S13i,j).

Finally, biosafety evaluation confirmed clinical compatibility of PADVs (Figure S14a). Throughout the four‐dose treatment regimen, body weight and temperature remained stable (Figure S14b,c), with no treatment‐related abnormalities observed in serum biochemical parameters or histology of major organs (Figure S14d,e).

In summary, PADVs represent a versatile nanotherapeutic strategy that achieves potent antitumour activity while effectively controlling metastatic progression.

## 3 Discussion

Immunotherapy aims to reactivate anti‐tumour immune cells and overcome tumour immune evasion mechanisms (Wang et al. 2022). However, its efficacy is profoundly influenced by the genetic landscape of tumour cells and the functional state of tumour‐infiltrating lymphocytes (Zhang and Zhang 2020; Morad et al. 2021; de Visser and Joyce 2023). A fundamental challenge faced by current approaches lies in a disconnection between inducing immune activation and sustaining it within a hostile TME (Morad et al. 2021; Ding et al. 2022). While conventional strategies often target individual components of tumour immune evasion, they typically fail to concurrently disrupt the immunosuppressive network and ignite robust immunogenic signalling. To address this gap, we developed PADVs, a biomimetic platform engineered to execute a ‘defense conversion’ strategy that repurposes tumour‐derived resistance mechanisms into therapeutic assets. This strategy operates through two mutually reinforcing mechanisms for augmenting the intrinsic anti‐tumour capabilities, thereby eradicating tumour, reducing recurrence and metastasis. Firstly, membrane‐anchored PD‐1 and SIRPα sequester tumour‐expressed PD‐L1 and CD47, reversing T cell exhaustion and myeloid suppression. Secondly, targeted co‐delivery of decitabine and LPS overcomes epigenetic silencing of GSDMD, reinstating pyroptosis‐mediated immunogenic cell death. This process not only eliminates tumour cells but, more importantly, generates an explosive release of tumour antigens and inflammatory mediators, establishing a positive feedback loop that propels DC maturation, CTL priming and memory T cell formation.

Tumours evolve through accumulated mutations that drive intricate pathogenesis (Hanahan 2022; Hanahan and Weinberg 2011). The interpatient, intertumour and intratumour heterogeneity significantly restricts the applicability of anti‐tumour drugs that target specific tumour‐associated antigens (TAAs) (van Weverwijk and Visser 2023). In this study, PADVs overcome this limitation by activating GSDMD to induce pyroptosis and expose individualized antigens, thereby eliciting highly tumour‐specific immune responses. Moreover, PADVs bypass tumour resistance to pyroptosis through demethylation‐mediated reactivation of gasdermin expression (Zhang et al. 2020). While developing an integrated nanoplatform that simultaneously enhances cytotoxic T cell responses and remodels the immunosuppressive TME is conceptually attractive, we acknowledge that tumour heterogeneity may demand patient‐specific therapeutic balances. The integration of dual functionalities within a single nanovesicle offers distinct advantages over conventional combination therapies. The synergistic targeting mechanism enhances tumour‐selective delivery of pyroptosis‐inducing cargo, minimizing off‐target exposure and mitigating systemic toxicity. Co‐loading both agents within the same nanovesicle ensures synchronized spatiotemporal delivery and coordinated action, creating the precise intracellular conditions necessary for pyroptotic cell death. With separate administration, the asynchronous pharmacokinetics of two free drugs would likely result in suboptimal timing between epigenetic priming and inflammasome activation, potentially compromising therapeutic efficacy (Mahoney et al. 2015). Importantly, PADVs represent a customizable platform that retains the flexibility to accommodate patient‐specific therapeutic needs. The membrane coating ratio and drug loading density can be adjusted to tailor the balance between checkpoint blockade intensity and pyroptosis potency for different tumour types or individual patient profiles, while membrane components can be derived from different immune cell sources to fine‐tune targeting specificity or immunomodulatory properties. By reshaping the host immune landscape, PADVs establish self‐sustaining immunity characterized by antigen‐specific memory T cells that confer durable protection against tumour recurrence and metastasis. This broad‐spectrum efficacy, combined with minimal off‐target toxicity, underscores the translational value of GSDMD as a master regulator of immunogenic cell death and positions pyroptosis induction as a scalable strategy against heterogeneous solid tumours (Fontana et al. 2024).

The architectural design of PADVs draws inspiration from extracellular vesicles (EVs) while extending their capabilities through rational engineering (Zhou et al. 2020; Li et al. 2024; Kwa et al. 2026). EVs possess inherent advantages in pharmacokinetic optimization and targeted biodistribution, having demonstrated unique advantages in tissue regeneration, immune regulation or tumour therapy (Wiklander et al. 2019; Dou et al. 2020; Zhang et al. 2021). The hybrid membrane shell preserves endogenous targeting and signalling functions, facilitating dual accumulation in tumours and lymphoid organs while minimizing immunogenicity (Cecchin et al. 2023; Xu et al. 2022). Complementarily, the MSN core enables esterase‐responsive payload release, ensuring precise subcellular activation of the pyroptotic cascade. This synergistic integration of biological targeting and controlled drug delivery distinguishes PADVs from conventional nanotherapies (Yang et al. 2024), allowing a minority of pyroptotic cells (≈20%) to produce disproportionately potent antitumour effects (≈90% suppression), an amplification effect critical for durable immune memory.

While this study represents a paradigm shift in harnessing nanotechnology for tumour immunotherapy, several translational aspects warrant further investigation. Dose optimization and combination regimens with modalities such as mRNA vaccines or oncolytic viruses may enhance response depth and durability. Understanding the spatiotemporal dynamics of immune memory formation following pyroptosis‐induced activation will also be crucial for clinical scheduling. Additionally, developing localized intervention strategies capable of initiating systemic immune defense represents a promising direction for future research. Overall, this platform provides a clinically translatable blueprint for next‐generation immunotherapy, and its integration with advanced bioengineering and computational modelling will accelerate the development of transformative immunotherapeutic platforms.

## 4 Methods

### 4.1 Cell Culture and Treatments

T cells were isolated from murine spleen as previously reported (Dou et al. 2020). CD3ε antibody (100314, BioLegend, 2 µg/mL) was first coated on the 6‐well plate at 37°C for 1 h. The spleen was isolated, homogenated and filtered to generate the single‐cell suspension. After erythrocyte lysis with ACK lysis buffer (C3702, Beyotime), T cells were collected and suspended with RPMI‐1640 medium (10% FBS, 1% penicillin/streptomycin). CD28 antibody (16‐0281‐85, eBioscience, 4 µg/mL) was used for activation (at least 48 h). IFN‐γ (PeproTech, 315‐05, 2 µg/mL) was further added to stimulate T cells for 24 h.

Macrophages were isolated from murine bone marrow, which were maintained in DMEM medium (High glucose, 10% FBS, 1% penicillin/streptomycin). The bone marrow single‐cell suspension was obtained from femurs and tibias as previously (Dou et al. 2020), and seeded in a 6‐well plate supplemented with M‐CSF (PeproTech, 315‐02, 20 ng/mL) for 7 days to induce mature bone marrow‐derived macrophages (BMDMs). LPS (Sigma–Aldrich, L3024, 1 µg/mL) was further added to stimulate macrophages for 24 h.

Fibroblasts were obtained from the murine dermis. The dermis was separated from the skin by incubation in dispase at 4°C overnight. The dermis was cut into small pieces and incubated with type I collagenase for 90 min at 37°C. The suspension was filtered and centrifuged at 800 rpm for 5 min to concentrate the cells. The cells were maintained in α‐MEM medium (10% FBS, 1% penicillin/streptomycin) at 37°C to generate fibroblasts.

B16F10, 4T1, CT26 and 293T cells were obtained from Procell (Wuhan, China). MC38 was obtained from American Type Culture Collection (ATCC). B16F10‐luciferase (FH1123) was purchased from FuHeng (Shanghai, China). CT26‐luciferase (iCell‐0061a) was purchased from Cellverse (Shanghai, China).

In the checkpoint blockade assay in vitro, various agents (50 µg/mL) were added into the medium for 24 h. In the pyroptosis induction assay in vitro, various agents (100 µg/mL) were added into the medium for 48 h. LPS (1 µg/mL) was used as control.

### 4.2 Synthesis of MSN^L@D^

MSNs were prepared by the classical CTAB‐templated, base‐catalyzed sol‐gel method according to the previous work (Dou et al. 2020). Then, 10 mg MSNs, 500 µg LPS (HY‐D1056, MedChemExpress), 600 µg decitabine (Dec, HY‐A0004, MedChemExpress) and 10 µL glycerol dimethacrylate (GDMA, 436895, Sigma–Aldrich) were completely dispersed in 50 µL water by ultrasonic treatment for 20 min. The mixture was placed in a vacuum oven and repeated vacuum (−1 bar) three times for 10 min each to generate LPS, Dec, and GDMA co‐loaded LPS@Dec‐MSN. At the same time, 24 mg APS was dissolved in 3 mL water and deoxidized for 20 min under nitrogen, 80 µL TEMED was dissolved in 800 µL water. After that, 2500 µL of APS solution and 440 µL of TEMED solution were mixed with LPS@Dec‐MSN. The mixture was stirred at room temperature under nitrogen for 1 h to form the PGD cap. The resulting nanoparticles were washed with water and dried in a vacuum oven at 37°C overnight, finally generating MSN^L@D^. The loading efficiency of MSN to LPS and Dec was calculated as follows.

$$Encapsulation\;efficiency=\frac{Weight\;of\;drug\;in\;MSNs}{Initial\;weight\;of\;drug\;}$$

The assembly process of MSN^L@D^ was characterized by the UV‐Vis spectra (GENESYS^TM^150, USA) with dilute aqueous solution in a 2 mm thick quartz cell. The chemical composition of MSN^L@D^ was measured by XPS (ESCALAB Xi^+^, USA). MSN and MSN^L@D^ before and after treatment by esterase were detected by TGA (STA449F5, DE). The pore size of these samples was measured by a surface area and porosity analyser (V‐Sorb 4800, Gold APP). The particle size and zeta‐potential were characterized by DLS (Zetasizer Nano ZSE, Malvern, UK). FITC‐labelled LPS (FITC‐LPS) was purchased from Sigma–Aldrich (F8666). Rhodamine (RhB) was selected as hydrophilic small‐molecule compound to replace Dec. FITC‐LPS and RhB were used to conduct fluorescence colocalization experiments.

### 4.3 Fabrication of Decoy Membrane and PADVs

IFN‐γ‐treated T cells and LPS‐treated macrophages were harvested. Cell membranes were isolated via hypotonic treatment followed by sonication to remove intracellular contents. An equal‐proportion mixture of T cell membranes and Mφ membranes was sequentially extruded through the 1000‐nm, 800‐nm and 400‐nm polycarbonate filters using a liposome extruder to generate decoy membranes (20 cycles per pore size). PADVs were constructed by co‐extruding the decoy membrane‐MSN mixture through the 200‐nm polycarbonate filters for 20 cycles. The resulting PADVs were purified by centrifugation at 6000 × *g* for 15 min. Membrane coating efficiency was quantified by measuring residual fluorescence intensity in the supernatant before and after PADV fabrication. TEM and DLS were used to analyse the morphology and size distributions of MSNs and PADVs. Membrane protein retention was verified by Coomassie blue staining (P0017, Beyotime) and Western blot.

The storage stability assay was detected by DLS (Malvern, UK). MSNs and PADVs were resuspended in water and PBS at 1 mg/mL to monitor the size variations for 7 days. Additionally, lyophilized PADVs were rehydrated after 7 days to measure size and zeta‐potential. The circulation stability was determined by serum stability and blood compatibility. The PADVs were resuspended in 100% FBS at 0.1 mg/mL, with absorbance at 560 nm measured at 20 min. Besides, PADVs at varying concentrations were incubated with RBC suspensions at 4°C for 24 h. The hemolysis was determined by UV‐Vis analysis. The cytotoxicity assay was performed by incubation of PADVs with fibroblasts and 293T cells for 24 h. Cell viability was determined by CCK8 kit (40203ES60, YEASEN).

### 4.4 Pyroptosis Detection In Vitro

Pyroptosis of tumour cells induced by PADVs was evaluated using complementary assays: CCK‐8 cell viability assay (40203ES, YEASEN), calcein AM release assay (40719ES, YEASEN), and lactate dehydrogenase (LDH) release assay (40209ES, YEASEN), following the manufacturers’ protocols. Briefly, tumour cells were seeded in 96‐well plates at 1 × 10^4^ cells/well. After 12 h, the cells were incubated with LPS (1 µg/mL), PADVs*^0^*, PADVs^L^ and PADVs^L@D^ (100 µg/mL) for 48 h. Cell morphology was captured by an inverted microscope (Olympus), and cell viability was quantified via the CCK8 kit. For LDH release detection, culture supernatants were collected after centrifugation at 800 × *g* for 5 min. LDH assay reagent was added to the supernatant, and the mixture was incubated at 37°C for 30 min before measuring OD_490_. The LDH release ratio was calculated relative to the positive control group.

For the calcein AM release assay, tumour cells were preloaded with calcein AM (4 µg/mL) at 37°C for 30 min, then washed with PBS to remove unincorporated dye. Cells were treated with the aforementioned agents for 48 h, as described. Calcein release into the supernatant was measured using a multimode plate reader (HH3400, PerkinElmer) (Ex485nm/Em530nm).

### 4.5 Animals and In Vivo Tumour Model Establishment

All C57BL/6J mice and Balb/c mice (6–8 weeks old) were obtained from the Animal Laboratory Center of the Fourth Military Medical University. B6.SJL‐Ptprca Pepcb/BoyJ mice (8 weeks old) were purchased from the Huachuang Sino Pharmaceutical Technology Co., Ltd (Jiangsu, China). Mice were housed under specific pathogen‐free (SPF), constant‐temperature conditions with ad libitum access to food and tap water. All animal experiments were approved by and conducted in accordance with the Guidelines of the Animal Care Committee of the Fourth Military Medical University.

The primary subcutaneous tumour model was established by inoculating 2 × 10^6^ tumour cells into the left hind leg dorsal region of mice. After 6 days (designated as Day 0), mice were randomly assigned to four treatment groups: intravenous injections of PADVs, PADVs^L^, or PADVs^L@D^ (10 mg/kg, 4 doses total) or an equal volume of PBS as a control. Tumour volume was measured using a vernier caliper and calculated as π/6 × length × width^2^. At Day 14, tumours and major organs were harvested for subsequent analyses, and survival was monitored until Day 30.

A tumour rechallenge model was established by subcutaneously inoculating 5 × 10^5^ tumour cells into the left hind leg dorsal region at Day 0. Mice received designated treatments at Days 3, 6 and 9, followed by surgical resection of primary tumours at Day 10. At Day 30, mice were subcutaneously rechallenged with 2 × 10^6^ B16F10 or MC38 cells in the contralateral region, and tumour growth was monitored until Day 50. For adoptive T cell transfer assays, spleen T cells were isolated from treated B16F10‐bearing donor mice at Day 10. A separate cohort of mice was inoculated with tumour cells at Day 5 to establish subcutaneous tumours. These recipient mice then received intravenous injections of T cell suspensions (1 × 10^6^ cells/mL, 100 µL per dose) at Days 12, 15 and 18, with tumour growth monitored until Day 25.

A tumour metastasis model was established by intravenously injecting 1 × 10^5^ luciferase‐B16F10/CT26 cells at Day 0 into mice pre‐inoculated subcutaneously with 1 × 10^6^ homologous cells 6 days earlier. Mice received intravenous treatments with various formulations (10 mg/kg, 3 doses total). Bioluminescence imaging was performed at Day 7 (B16F10‐luc) or Days 7, 14 and 21 (CT26‐luc) using D‐Luciferin potassium salt (HY‐12591B, MedChemExpress). Primary tumours and metastatic lungs were harvested at Day 14 (B16F10) or Day 21 (CT26) for further analysis, and survival was tracked until Day 40.

For immune microenvironment investigations, subcutaneous tumour models were established as described above, with concurrent transplantation of allogeneic inguinal adipose tissue at the same site. After 14 days, tumours and adipose tissues were collected for histological, flow cytometric or Western blotting analyses. Additionally, subcutaneous tumour‐bearing mice received intravenous injections of anti‐mouse PD‐L1 antibody (BE0101, BioXcell; 100 µg per mouse per dose) for a total of three doses.

### 4.6 RNA‐seq Analysis

Tumour tissue in each group was collected to extract total RNA for RNA sequencing analysis provided by LC‐Bio Technology CO., Ltd. (Hangzhou, China). To identify DEGs across different treatment groups, we leveraged the Wilcoxon test, stringently adjusting *p* values for false discovery rate (FDR) to mitigate false positives. Genes satisfying the criteria of FDR < 0.05 and log_2_ fold‐change (|log_2_ FC|) > 1 were deemed significant. Subsequently, we delved into the functional landscape of these DEGs through GO enrichment analysis, utilizing the clusterProfiler and enrichplot R packages. Only GO terms with FDR < 0.05 were considered statistically significant. To visualize the enriched GO terms and their associated DEGs, we generated heatmap plots.

### 4.7 Statistical Analysis

Data are presented as the means ± SD of at least triplicate independent replicates. Statistical significance was determined using two‐tailed unpaired Student's *t*‐test, one‐way ANOVA, Mann–Whitney test, or Log‐rank test, with Tukey or Dunnett T3 post hoc tests applied following one‐way ANOVA. Statistical parameters are specified in the figure legends. Differences between groups were defined as non‐significant (n.s.) when *p* > 0.05 and statistically significant when *p* < 0.05, with exact *p* values marked on the figures. Statistical analyses were performed using SPSS 19.0 (IBM, USA), and graphing was conducted using GraphPad Prism 9.00 (GraphPad Software, USA).

## Author Contributions

**Geng Dou**: conceptualization, methodology, software, data curation, supervision, formal analysis, validation, investigation, funding acquisition, visualization, project administration, writing – original draft, writing – review and editing. **Jiani Liu**: methodology, software, data curation, project administration, formal analysis, validation, visualization, investigation, writing – original draft. **Ran Tian**: conceptualization, methodology, software, data curation, formal analysis, project administration, resources, writing – original draft, investigation, validation, visualization. **Keying Zhang**: conceptualization, methodology, software, data curation, formal analysis, funding acquisition, investigation, validation, visualization, writing – original draft, project administration, resources. **Meng Suo**: methodology, data curation, resources, project administration, visualization, investigation, formal analysis. **Ding Zhou**: methodology, investigation, visualization, formal analysis, project administration, resources, data curation. **Yipeng Zhang**: investigation, methodology, visualization, project administration. **Xuemei Liu**: investigation, methodology, visualization, project administration. **Xinyu Qiu**: investigation, funding acquisition, methodology, visualization, formal analysis. **Jinxin Kuang**: investigation, methodology, visualization, formal analysis. **Zhaonan Zou**: investigation, methodology, visualization, formal analysis. **Shuchang Wang**: investigation, validation. **Lu Zhao**: investigation, methodology, visualization, formal analysis. **Feng Ding**: investigation, methodology, visualization, formal analysis. **Di Wang**: investigation, methodology, formal analysis, data curation, writing – review and editing, visualization. **Congcong Guan**: investigation, validation. **Xiaoyu Zhu**: investigation, methodology, visualization, resources, funding acquisition. **Dongyao Wang**: investigation, methodology, visualization, formal analysis. **Siying Liu**: investigation, methodology, visualization, formal analysis. **Tianfu Zhang**: investigation, validation, methodology, visualization, formal analysis, supervision, data curation, writing – review and editing. **Yimin Zhao**: conceptualization, methodology, validation, visualization, investigation, formal analysis, project administration, supervision, data curation. **Shiyu Liu**: conceptualization, methodology, data curation, formal analysis, validation, investigation, funding acquisition, writing – review and editing, project administration, supervision.

## Conflicts of Interest

The authors declare no conflicts of interest.

## Supporting information




**Supporting Information** jev270356‐sup‐0001‐SuppMat.docx

## Data Availability

All the essential data are included in the main text and supplementary materials. The data and materials used in this study are available from the corresponding authors upon reasonable request.
