# Supplementary material for: Pyroptosis‐Amplified Decoy Nanovesicles Orchestrate Pyroptotic Antigen Explosion and Endogenous Immune Revitalization for Potentiating Tumour Immunotherapy
Source: J Extracell Vesicles. 2026 Aug 28;15(9):e70356. doi: 10.1002/jev2.70356 (PMC13522832; doi:10.1002/jev2.70356)
Supplement: Supplementary file 1 [file JEV2-15-e70356-s001.docx]

Supplementary Materials for

**Pyroptosis-Amplified Decoy Nanovesicles Orchestrate Pyroptotic Antigen Explosion and Endogenous Immune Revitalization for Potentiating Tumor Immunotherapy**

Geng Dou^1,2#,*^, Jiani Liu^3#^, Ran Tian^4#^, Keying Zhang^5#^, Meng Suo^6^, Ding Zhou^7^, Yipeng Zhang^1,2^, Xuemei Liu^8^, Xinyu Qiu^1^, Jinxin Kuang^1^, Zhaonan Zou^1^, Shuchang Wang^1^, Lu Zhao^9^, Feng Ding^1,2^, Di Wang^1,10^, Congcong Guan^1^, Xiaoyu Zhu^11^, Dongyao Wang^11^, Siying Liu^1,2^, Tianfu Zhang^6*^, Yimin Zhao^1*^, Shiyu Liu^1*^

Correspondence to: liushiyu@vip.163.com; zhaoym@fmmu.edu.cn; doug0714@163.com; zhangtf@gzhmu.edu.cn.

**This PDF file includes:**

Materials and Methods

Figures. S1 to S14

Tables S1

**Materials and Methods**

**Bioinformatic analysis of melanoma based on ESTIMATE and TIMER database**

We retrieved comprehensive skin cutaneous melanoma data from ESTIMATE database (<https://bioinformatics.mdanderson.org/estimate>) and Tumor Immune Estimation Resource database (TIMER, <http://timer.cistrome.org>). To evaluate the tumor microenvironment (TME), we employed the ESTIMATE algorithm to quantify tumor purity and assess the infiltration of stromal and immune cells. Specifically, the ImmuneScore and StromalScore, positively correlated with immune and stromal content, respectively, were calculated. The ESTIMATEScore, their sum, provided an integrated measure of TME composition.

To refine our analysis, we utilized the MCP-counter algorithm and CIBERSORT method in the TIMER database to precisely quantify the abundance of infiltrating immune and stromal cell subsets within melanoma. We focused on quantifying key immune cell types, including T cells, CD8^+^ T cells, and cytotoxic lymphocytes (CTLs). Then, patients were stratified into High and Low groups according to the median level of TME components, facilitating survival analysis using Kaplan-Meier curves to assess prognostic significance. The influence of TME on clinicopathological characteristics was analyzed using the Wilcox test. Furthermore, univariate and multivariate Cox regression analyses were performed to identify independent prognostic factors in the melanoma cohort retrieved from TIMER database. Subsequently, we mined the Gene Expression Profiling Interactive Analysis (GEPIA) website (http://gepia.cancer-pku.cn/) for the expression levels of GSDMD in melanoma versus normal tissue, along with its prognostic impact on overall and disease-free survival. Additionally, we explored the correlation of GSDMD with markers of effector T cells (CX3CR, FGFBP2, and FCGR3A), effector memory T cells (PDCD1, DUSP4, GZMK, GZMA, and IFNG), and central memory T cells (CCR7, SELL, and IL7R) by using GEPIA. The study’s workflow was visually summarized in Fig. 1a.

**Single cell analysis**

We obtained the GSE215120 dataset from Gene Expression Omnibus (GEO) and focused our analysis on single cell sequencing profiles from untreated melanoma primary tissues. Utilizing R (v4.2) and Seurat (v4.3.0), we conducted a meticulous data quality control process, retaining 81,583 cells that met stringent criteria: expressing > 200 genes, possessing > 300 unique molecular identifiers (UMIs), and exhibiting < 20% mitochondrial gene expression. After down-conversion analysis using TSNE, we categorized the cells based on their marker gene expression profiles, distinguishing melanoma cells (MLANA, PMEL, MITF, and DCT), endothelial cells (VWF and PECAM1), fibroblasts (COL1A1 and COL3A1), T cells (CD3D and CD3E), natural killer cells (FGFBP2 and KLRD1), B cells (MS4A1 and CD79A), and myeloid cells (CD68 and CD14). Furthermore, we mapped the expression of GSDMD in these different cell types. To gain insight into the underlying biological pathways, we employed the clusterProfiler package (v4.6.2) for enrichment analysis and visualized the top 10 pathways. Intercellular communication analysis was performed using CellChat (v1.6.1) to investigate receptor-ligand interactions between CD8^+^ T cells and GSDMD^+^ melanoma cells, providing novel insights into the immune microenvironment dynamics within melanoma.

**Mendelian randomization analysis**

Two-sample Mendelian randomization was performed using large genome-wide association study (GWAS) genetic data to assess causal effects between peripheral immunophenotypes and melanoma risk. The genetic variation of each immune trait was obtained from the GWAS catalog (registration numbers GCST0001391 to GCST0002121), which is the largest GWAS published so far on peripheral blood immune phenotypes. Melanoma GWAS datasets were obtained from the IEU OPEN GWAS (https://gwas.mrcieu.ac.uk/), including ieu-b-4969, ukb-d-C3-MELANOMA-SKIN, ukb-b-12915, and ukb-d-C43. Inverse variance weighted method was adopted as the primary approach, aiming to robustly estimate causal effects while accounting for potential biases. Cochran’s Q test was performed to evaluate the consistency of our findings across studies, mitigating concerns over outcome heterogeneity. To further consolidate our insights, we synthesized the evidence by identifying consistently involved immune cell subsets across multiple GWAS databases and visualized them using Venn diagrams. This approach not only strengthened our conclusions but also illuminated common denominators in the immunological landscape of melanoma risk.

**In vitro esterase-responsive drug release of MSN^L@D^**

The *in vitro* drug release process was determined by the UV absorption intensity change of the solution over incubation time using UV-Vis spectrophotometer (GENESYS^TM^150, USA). In brief, MSN^L@D^ was dispersed in PBS at 1 mg/ml with or without esterase under shaking at 37 °C. Then the supernatant was collected by centrifugation at different timepoints. The UV absorption intensity of the supernatant was tested to monitor the *in vitro* drug release. The drug release from MSN^L@D^ in water, PBS and serum was detected as above.

**Fluorescence Resonance Energy Transfer (FRET) Assay**

To evaluate the functional fusion between T cell membranes and macrophage membranes, we employed fluorescence resonance energy transfer (FRET) to monitor the spatial proximity of fluorescently labeled molecules on the two types of membranes. Briefly, T cell membranes and macrophage membranes were isolated as described previously and subsequently labeled with DiO (40725ES, YEASEN) and DiI (40726ES, YEASEN), respectively. These labeled membranes were then fused in varying ratios of T cell membrane to macrophage membrane (Tm : Mφm = 1:0, 5:1, 4:1, 3:1, 2:1, 1:1) to form hybrid membranes (Hm). The resulting products were analyzed using fluorescence spectroscopy with an excitation wavelength of 484 nm, focusing on the emission peak at 567 nm. A simple mixture of the two labeled membranes without fusion served as a control.

**Stability analysis**

To assess storage stability, MSNs and PADVs were resuspended in water and PBS to measure the size change for 1 week. The size and zeta-potential of PADVs before lyophilization and after resuspension were also measured by DLS.

**TEM**

MSN, MSN^L@D^ and PADVs^L@D^ were suspended in deionized water at 0.5 mg/ml. 4 μl suspension was dropped on the 200-mesh carbon-free copper grid for 10 min. PADVs^L@D^ sample needed to be stained with 2% phosphotungstic acid for 60 sec. After washing with distilled water and air-drying, the samples were imaged by TEM (TECNAI Spirit, FEI) at 120 kV. For the immunogold electron microscopy, different agents (0.5 mg/ml, 4 μl) were dropped on the 200-mesh carbon-free copper grid for 10 min. After washing and blocking with 5% BSA, the grid was incubated with primary antibodies (1:20) for 60 min at room temperature. After washing with distilled water, the grid was treated with 10-nm anti-rat IgG gold secondary antibody and 25-nm anti-rabbit IgG gold secondary antibody (Electron Microscopy Sciences) for 1 h at room temperature. After rinsing with distilled water, the grid stained with 2% phosphotungstic acid for 30 s. After rinsing in distilled water and air-dying, all grids were imaged by TEM.

**Cell binding assay**

To assess the capacity of PADVs to interact with tumor cells via checkpoint molecules, B16F10/4T1/CT26 cells were seeded into 35 mm glass-bottom dishes at a density of 2 × 10⁵ cells per dish and allowed to adhere until they reached 50%-60% confluence. After washing three times with PBS, the tumor cells were incubated with RhB-labeled MSN or PADVs for 2 h at 4 °C. For competitive binding assay, recombinant PD-L1 protein (20 μg/mL, ab221310, Abcam) was preincubated with PADVs for 4 h before co-incubation with tumor cells. After incubation, unbound MSN or PADVs were removed by washing three times with PBS. The tumor cells were then stained with the cell membrane probe PKH67 (MINI67, Sigma Aldrich) and observed under a confocal microscope (Nikon, Japan).

**T cell co-incubation assay**

To prove that PADVs can block the destruction mechanism of tumor cells on T cells, the following experiments were designed to detect T cell function. T cells were isolated from the mice spleen and activated for 48 h. B16F10 cells were incubated MSN/TNVs/MφNVs/PADVs (50 μg/ml) for 48 h. The B16F10 cells were collected and co-incubated with activated T cells for 24 h. Then, the T cells were obtained to assess the proliferation ability by EdU and CD25 staining. The positive population was analyzed by fluorescence imaging and flow cytometry. In addition, B16F10 cells were seeded into the 96-well plate (1.5×10^4^ cells/well) and treated with different agents as above. After refreshing the medium, the isolated CD8^+^ T cells by CD8^+^ T Cell Isolation kit (480007, BioLegend) were added to B16F10 cells for 48 h. The cell viability of B16F10 cells was detected by CCK8 kit. Besides, T cells were directly incubated with PADVs*^0^*, PADVs^L^ and PADVs^L@D^ (50 μg/ml) for 24 h, and the proliferation ability was evaluated by CFDA SE cell proliferation and cell tracking kit follow the instruction (40714ES, YEASEN).

**Dendritic cell activation test in vitro**

The co-culture system of dendritic cells and tumor cells is based on 0.4-μm transwell (3412, Corning), with B16F10 cells in the top chamber and DCs in the bottom chamber. B16F10 cells were treated with different agents (100 μg/ml) for 24 h. Then, DCs were obtained to detect the level of CD80 and CD86 by flow cytometry. And supernatants were collected and purified by centrifugation to measure the cytokines secreted from DCs by ELISA. Untreated and LPS-stimulated DCs were served as the negative and positive control.

**Phagocytosis of tumor cells by macrophages in vitro**

Firstly, the tumor cells (B16F10, 4T1, CT26) were cultured in the 6-well plate and incubated with various agents (50 µg/ml) for 24 h. Then, the prepared BMDMs and tumor cells were collected and stained with DiD (40758ES, YEASEN) and DiO (40725ES, YEASEN), respectively. The DiD-labelled BMDMs and DiO-labelled tumor cells (1 : 1) were co-incubated at the glass-bottom dishes for 8 h. The fluorescence images were taken by confocal laser scanning microscopy (CLSM) (Nikon). Phagocytosis ratio was measured as the red signaling in the green signaling.

**T cell co-stimulation activation test**

To evaluate the interaction between PADVs and T cells, T cells were incubated with 100 μg/mL PADVs for different time points. The interaction mode was visualized by staining the actin cytoskeleton with FITC-phalloidin (40735ES, YEASEN) to observe the spatial association between PADVs and T cells.

To assess whether PADVs induce pyroptosis in T cells, T cells were incubated with 100 μg/mL of PADVs*^0^*, PADVs^L^, PADVs^L@D^ for 24 h. Pyroptosis was evaluated by flow cytometry to detect propidium iodide (PI)-positive cells (40755ES, YEASEN), and western blot analysis was performed to assess GSDMD cleavage. T cell proliferation was assessed using a CFSE-based proliferation kit following co-incubation with the different PADV formulations (YEASEN, 40714ES). To determine whether the T cell response was mediated by co-stimulatory molecules, PADVs were pre-incubated with blocking antibodies against mouse CD40 (BE0016-2, BioXcell) or mouse CD86 (BE0025, BioXcell) at 4°C for 24 h prior to co-incubation with T cells. T cell activation and proliferation were then analyzed as described above.

**Tumor targeting and permeability assay**

The penetration ability of PADVs into tumor deeply was tested in vitro by cultivating 3D tumor cell spheroids. The 96-well plate was firstly coated with the matrigel (354234, Corning) at 37 °C for 30 min. Then, the B16F10 cells were collected and seeded into the plate at 100 cells/well. After incubation for 3-5 days, the 3D multicellular spheroids were formed. The formed 3D multicellular spheroids were treated with various vesicles agents with the concentration of 50 µg/ml, MSN served as control. After 48 h, the 3D multicellular spheroids were fixed with 4% PFA solution and stained with Hoechst. The infiltration of different agents into the spheroids were determined using layer scanning on the z-axis by confocal microscopy (Nikon).

The subcutaneous tumor model was established as above. After 12 days, the mice with the tumor volume reached 120~200 mm^3^ were included into the experiment. MSN labelled with RhB or DiR was prepared and used to fabricate fluorescence-labelled PADVs. RBCNVs were prepared using the same protocol. DiR-labelled MSN/PADVs/RBCNVs were injected into tumor-bearing mice intravenously at 10 mg/kg. In vivo fluorescence imaging was performed to determine the distribution of different agents in tumor region using an *in vivo* imaging system (IVIS) (Perkin Elmer) at 24 h. Likewise, RhB-labelled MSN/PADVs/RBCNVs were injected into the mice. The blood, tumor, lymph nodes and major organs were collected to measure the distribution level by fluorescence detection.

**Proteomic analysis of protein corona formation on PADVs**

To investigate the potential impact of protein corona formation on PADVs upon exposure to biological fluids, we performed comparative proteomic analysis between naked PADVs and plasma-incubated PADVs. In brief, 500 μg of PADVs were incubated with 500 μL of EV-depleted fetal bovine serum (FBS) for 1 hour at 37°C with constant shaking at 300 rpm. After incubation, the mixture was centrifuged at 6000 g for 15 min to pellet the PADVs, and the resulting FBS-incubated PADVs (FBS-PADVs) were collected. Both naked PADVs and FBS-PADVs were then subjected to proteomic analysis by liquid chromatography-mass spectrometry (LC-MS). To focus specifically on the protein corona derived from FBS exposure, the acquired protein profiles were searched against a bovine protein database, thereby excluding all mouse-derived proteins (originating from the PADV membrane) and enabling specific identification of adsorbed plasma proteins on the PADV surface. The proteomic analysis was performed by Shanghai Bioprofile Biotechnology Co., Ltd.

**Western blotting**

For the cell samples, the protein was collected by RIPA lysis buffer (P0013B, Beyotime). The membrane protein proportion was obtained by membrane and cytosol protein extraction kit (20127ES50, YEASEN). For the tissue samples, the tissues were rinsed and homogenized, then the tissue protein were collected. The protein samples were prepared by the BCA kit and loading buffer at a concentration of 1 μg/μl. Electrophoresis and transfer operation was conducted using the Bio-Rad system. PVDF membrane was blocked with 5% BSA solution, and then was incubated with the primary antibodies at 4℃ overnight. After incubation with the HRP-conjugated secondary antibodies for 1 h at room temperature, the PVDF membranes were imaged under the imaging system (Tanon 4600) with hypersensitive ECL chemiluminescence kit (AP0081, AccuRef Scientific). The antibodies used are listed in Supplementary Table S1.

**Immunohistochemistry**

The paraffin-embedded sections were deparaffinized in xylene and conducted gradient dehydration as the instruction. After antigen retrieval by heating the sections to 95°C in citrate buffer (pH=6.0, 20 min), sections were blocked with 3% BSA solution for 30 min at room temperature. The sections were incubated with the primary antibody overnight at 4°C. The sections were washed with TBS and incubated with HRP-conjugated secondary antibody for 30 min at room temperature, followed by TBS washing and DAB incubation for 5 min. The sections were finally counterstained with haematoxylin and sealed with neutral balsam. The images were captured by the Olympus workstation. The antibodies used are listed in Supplementary Table S1.

**Immunofluorescence**

Cell samples were prepared by fixing the cells after treatments with 4% paraformaldehyde solution (PFA) solution (P0099, Beyotime) at 4℃ for 2 h. Tissue samples were prepared into the 10-μm section after fixation, dehydration and embedding. Then, the samples were permeated with 0.1% Triton X-100 for 15 min and blocked with goat serum at 37℃ for 30 min. After incubation with primary antibodies at 4℃ overnight, the samples were washed with PBS for 3 times and incubated with the fluorescence-conjugated secondary antibodies at 37℃ for 1 h. Finally, the samples were counterstained with Hoechst 33342 (B2261, Sigma-Aldrich) for 15 min, and were imaged by CLSM (Nikon). The antibodies used are listed in Supplementary Table S1.

Live and dead cell staining was performed by Calcein-AM/PI double stain kit (40747ES, YEASEN). Multicolor immunofluorescence was prepared and provided by ServiceBio (Wuhan, China).

**Flow cytometry**

Tissue samples were obtained at the timepoint and homogenized to obtain the single-cell suspension. The cell samples were also collected and washed with cold PBS to generate single-cell suspension. For detection of cell surface molecules, the samples were incubated with the antibodies (1:50) at 37℃ for 30 min in dark. For detection of intracellular molecules, the samples were treated with the intracellular fixation and permeabilization buffer (2472791, eBioscience), and incubated with the antibodies as above. Then, the cells were washed twice with PBS and filtered for detection by the flow cytometry (Beckman Coulter, USA). For the detection of cell viability, the PI solution was used follow the instruction (40710ES03, YEASEN). The results and graphs were processed using FlowJo_V10 software. The antibodies used are listed in Supplementary Table S1.

**ELISA**

Tumor samples collected at the determined timepoint were homogenized to obtain the tissue homogenate. The serum was collected from the whole blood by centrifugation (4000 rpm, 5 min). The cell supernatant was purified to remove the cell debris. The concentration of IFN-γ, TNF-α and IL-6 in the tissue homogenate, serum and supernatant was determined by the ELISA kit as the instructions (EMC101g(H).96, EMC102a.96, EMC004.96, NeoBioscience).

Figure. S1.

**Supplementary Figure S1**

**Prognostic relevance and causal association of T cell infiltration with melanoma.**

**a** Kaplan-Meier curves demonstrating the independent prognostic value of CTLs infiltration in melanoma, with fractions quantified using the MCPcounter algorithm in the TIMER database.

**b** Correlative analyses highlighting the clinical relevance of CTLs fractions in the melanoma cohort retrieved from TIMER database.

**c-d** Univariate and multivariate Cox regression models assessing the independent predictive power of CTLs on melanoma prognosis in the melanoma cohort retrieved from TIMER database.

**e** Mendelian randomization analysis elucidating the causal relationship between CD8^+^ T cell abundance and melanoma risk. SSC-A on HLA DR^+^ CD8br: Side Scatter Area of HLA-DR-Positive CD8bright T Cells. TD CD8br %CD8br: Percentage of Terminal Differentiated CD8bright T Cells among Total CD8bright T Cells. CM CD8br AC: Absolute Count of Central Memory CD8bright T Cells. CD8 on CD39^+^ CD8br: CD8 Expression Level on CD39-Positive CD8bright T Cells. HLA DR^+^ CD8br %T cell: Percentage of HLA-DR-Positive CD8bright T Cells among Total T Cells. HLA-DR⁺ CD8br AC: Absolute Count of HLA-DR-Positive CD8bright T Cells.

**f-k** Venn diagrams (f, i) identifying shared melanoma-associated CD8^+^ T cell subsets across independent datasets (ieu-b-4969/ukb-d-C3 for f, ukb-b-12915/ukb-d-C43 for i). Corresponding Mendelian randomization analyses (g, h for f; j, k for i) exhibiting the causal links between tumor-responsive CD8^+^ T cell subsets (TD CD8br %CD8br and HLA-DR^+^ CD8br AC, respectively) and melanoma.

**l** H&E and immunofluorescence analysis of the immune infiltrating levels in tumor and transplanted fat tissue. Scale bar, 20 μm.

**m** Flow cytometry of CD8^+^ T cells in tumor and transplanted fat tissue (n = 5).

**n** Western blotting analysis of PD-L1 level in skin and melanoma.

**o** Intercellular communication analysis between CD8^+^ T cells and GSDMD^+^ melanoma cells.

**p** Enrichment analysis of biological pathways modulated by GSDMD expression.

Exact *p* values were calculated using unpaired two-tailed Student’s t-test (m).

Figure. S2.

**Supplementary Figure S2**

**Prognostic significance of GSDMD and its correlation with T cell signatures across multiple tumors.**

**a-c** Kaplan-Meier curves demonstrating the independent prognostic value of GSDMD for OS and DFS in GEPIA-derived breast invasive carcinoma patients (a), renal papillary cell carcinoma patients (b) and rectum adenocarcinoma patients (c).

**d-f** Pearson correlation analysis of the relationship between GSDMD expression and the signatures of effector T cells, effector memory T cells in breast invasive carcinoma (d), renal papillary cell carcinoma (e) and rectum adenocarcinoma patients (f).

**g-h** Comparison of effector T cell (g) and naive T cell (h) abundance between tumor tissues and normal tissues in rectal adenocarcinoma, renal papillary cell carcinoma and breast invasive carcinoma.

**i** Comparison of GSDMD expression between tumor tissues and normal tissues in skin cutaneous melanoma.

**j** Comparison of GSDMD promoter methylation levels in skin cutaneous melanoma patients stratified by disease stage (N1 vs N3).

**k** Comparison of GSDMD promoter methylation levels in skin cutaneous melanoma patients across age groups (41-60 years vs 61-80 years).

**l** Comparison of GSDMD expression in skin cutaneous melanoma patients across age groups (41-60 years vs 61-80 years).

**m** Western blotting analysis of GSDMD level in various tumor and non-tumor cells.

**n** Western blotting analysis of GSDMD level in B16F10, 4T1 and CT26 cells after treatment of decitabine.

Figure. S3.

**Supplementary Figure S3**

**Construction process and characterization of PADVs.**

**a** X-ray photoelectron spectroscopy (XPS) of C 1s, N 1s, O 1s, P 2p and Si 2p regions of MSN^L@D^.

**b-c** The encapsulation efficiency of LPS/Dec on MSN.

**d** The release percentage of LPS or Dec from MSN^L@D^ when incubation in water, PBS or serum.

**e-f** Flow cytometric analysis (e) and fluorescence images (f) of CD45 and CD3 level in T cells. Scale bar, 50 μm.

**g-h** Flow cytometric analysis (g) and fluorescence images (h) of CD11b and F4/80 level in macrophages. Scale bar, 50 μm.

**i-k** Western blotting (i), flow cytometry (j) and fluorescence images (k) of PD-1 level in T cells simulated by IFN-γ. Scale bar, 50 μm.

**l-n** Western blotting (l), flow cytometry (m) and fluorescence images (n) of SIRPα level in macrophages simulated by LPS. Scale bar, 50 μm.

**o** Schematic diagram of the decoy membrane (Dm) preparation process.

**p** A simple mixture of the DiO-Tm and DiI-Mφm was detected by fluorescence spectroscopy at Ex484nm/Em567nm.

**q** The optimal ratio of membrane coating percentage determined by stability performance (n = 3).

**r** The membrane coating percentage of different formulations (n = 6).

**s** The expression of CD3 and F4/80 on surface of different nanovesicles were detected by immunoelectron microscopy. Scale bar, 50 nm.

**t** Western blotting analysis of the purity of PADVs.

**u** Size distribution of Dm, MSN and PADVs.

**v** Serum stability test of MSN and PADVs (n = 20).

**w** Cytotoxicity test of PADVs in fibroblast and 293T cells (n = 6).

Exact *p* values were calculated using unpaired two-tailed Student’s t-test (v).

Figure. S4.

**Supplementary Figure S****4**

**Extracellular LPS and decitabine (Dec) fail to induce pyroptosis in tumor cells.**

**a** Representative multi-cell fluorescence images showing PADVs-B16F10 cell interaction in the absence or presence of rPD-L1. Scale bar, 20 μm.

**b** Representative photos of tumor cells after treatment with LPS alone, decitabine (Dec) alone, or their combination for 24 h.

**c** Cell viability test of tumor cells after indicated treatments (n = 6).

**d** Western blotting analysis of GSDMD cleavage in tumor cells after indicated treatments.

**Figure. S5.**

**Supplementary Figure S5**

**PADVs-mediated immune checkpoint blockade and enhancement of macrophage phagocytosis in 4T1 and CT26 tumor cells in vitro.**

**a** Fluorescence visualization of PADVs-4T1 cell interaction. Scale bar, 20 μm.

**b-c** Flow cytometric quantification of PD-L1 (b) and CD47 (c) expression on 4T1 cells post-PADVs treatment.

**d** Western blotting of PD-L1 and CD47 level on 4T1 cell surface.

**e** Phagocytosis ratio of macrophages toward 4T1 cells treated with different formulations (n = 5). Scale bar, 100 μm.

**f** Fluorescence visualization of PADVs-CT26 cell interaction. Scale bar, 20 μm.

**g-h** Flow cytometric quantification of PD-L1 (g) and CD47 (h) expression on CT26 cells post-PADVs treatment.

**i** Western blotting of PD-L1 and CD47 level on CT26 cell surface.

**j** Phagocytosis ratio of macrophages toward CT26 cells treated with different formulations (n = 5). Scale bar, 100 μm.

Exact *p* values were calculated using one-way ANOVA with Tukey’s post hoc test (e, j).

Figure. S6.

**Supplementary Figure S6**

**PADVs^L@D^ induced pyroptosis of tumor cells in vitro.**

**a** Western blotting analysis of extracellular HMGB1 level after PADVs^L@D^ treatment.

**b** Western blotting analysis of caspase-11 activation and GSDMD cleavage in 4T1 and CT26 cells after different treatments.

**c** Cell viability test of 4T1 and CT26 cells after different treatments (n = 6).

**d** PI positive rate of 4T1 and CT26 cells analyzed by flow cytometry.

**e** Representative photos of 4T1 and CT26 cells after different treatments. Black arrow indicates typical pyroptotic cells.

**f** Live/dead cell staining (Calcein AM/PI) of 4T1 and CT26 cells after different treatments. Scale bar, 50 μm.

**g** LDH release assay of 4T1 and CT26 cells after different treatments (n = 6).

**h** Calcein release assay of 4T1 and CT26 cells after different treatments (n = 6).

**i** HMGB1 translocation imaging of 4T1 and CT26 cells after different treatments. Scale bar, 50 μm.

n.s., not significant. Exact *p* values were calculated using one-way ANOVA with Tukey’s post hoc test (c, g) or Dunnett T3 post hoc test (g, h).

Figure. S7.

**Supplementary Figure S7**

**PADVs directly activate T cells via surface co-stimulatory molecules without inducing pyroptosis.**

**a** Flow cytometric analysis of CD25^+^ activated T cell populations following co-culture with differentially treated B16F10 cells.

**b** High-resolution fluorescent images of PADVs-T cell interactions over time. Scale bar, 5 μm.

**c** Flow cytometry analysis of PI^+^ population in T cells treated with PADVs^L@D^ over time.

**d** Western blotting analysis of GSDMD cleavage in T cells after treatment with PADVs^L@D^ over time.

**e** Flow cytometry analysis of CFSE-labeled T cell proliferation with pre-incubation with anti-CD40/CD86 neutralizing antibodies (or isotype control).

Figure. S8.

**Supplementary Figure S8**

**Chemotactic properties of decoy membrane ensure tumor targeting capability of PADVs.**

**a** In vitro penetration ability of PADVs assessed by z-stack confocal microscopy in 3D cell spheroids. Scale bar, 50 μm.

**b** Pharmacokinetic analysis of in vivo circulation time for MSN, PADVs and RBCNVs (n = 6).

**c** In vivo fluorescence imaging for biodistribution of different formulations in tumor-bearing mice.

**d** Western blotting analysis of chemotactic molecule expression on the surface of different nanovesicles.

**e** Fluorescence images for the enrichment level of different formulations in tumor and major organs. Scale bar, 100 μm.

**f** Fluorescence images showing the infiltration of different nanovesicles in tumors and their spatial relationship with blood vessels. Scale bar, 100 μm.

**g** Time-dependent enrichment of PADVs in tumor. Scale bar, 100 μm.

Figure. S9.

**Supplementary Figure S9**

**Proteomic profiling of protein corona formed on PADVs after FBS incubation.**

**a** Volcano plot showing differentially expressed proteins between FBS-incubated PADVs (FBS-PADVs) and naked PADVs. Upregulated proteins (red, 405, 5.64%) and downregulated proteins (blue, 176, 2.45%) are indicated (|log₂FC| > 1, *p* < 0.05).

**b** Radar plot of differentially expressed proteins in FBS-PADVs vs naked PADVs group. The radial overlap reflects the preservation of endogenous membrane proteins, whereas the radial asymmetry in specific regions indicates the adsorption of serum-derived proteins.

**c** GO enrichment analysis of differentially expressed proteins, highlighting the top terms for biological process (BP), cellular component (CC), and molecular function (MF).

**d** Gene Ontology (GO) cellular component (CC) enrichment analysis of all differentially expressed proteins between FBS-PADVs and naked PADVs, showing enrichment in extracellular matrix, lipoprotein particles, and collagen-containing extracellular matrix.

**e** Gene Ontology (GO) biological process (BP) enrichment analysis of upregulated proteins only in FBS-PADVs, revealing significant enrichment in humoral immune response, complement activation, coagulation cascade, and negative regulation of proteolysis.

**f** KEGG pathway enrichment analysis of proteins enriched in FBS-PADVs, showing significant enrichment in complement and coagulation cascades, cholesterol metabolism, and ECM-receptor interaction.

**g** Coomassie Brilliant Blue staining of protein extracts from naked PADVs, FBS-PADVs, and FBS alone. A prominent band at ~66 kDa corresponding to albumin was observed in FBS-PADVs, confirming albumin as the major component of the protein corona.

Figure. S10.

**Supplementary Figure S10**

**PADVs^L@D^ demonstrates broad-spectrum anti-tumor efficacy in breast and colon cancer models.**

**a** Schematic of primary subcutaneous 4T1 breast tumor model establishment and therapeutic regimen.

**b** Gross morphology of tumors in each group. Scale bar, 1 cm.

**c-d** Tumor volume (c) and weight (d) in each group (n = 6).

**e** Tumor inhibition rate in each group (n = 6).

**f** Histopathological evaluation of tumor by H&E staining. Scale bar, 100 μm.

**g** Flow cytometric quantification of PI^+^ dead cells in tumor tissues.

**h** TUNEL and GSDMDC staining of tumor after treatment. Scale bars, 100 μm.

**i** Western blotting analysis of GSDMD cleavage in tumor.

**j** CD8^+^ T cell infiltration level quantified by immunofluorescence. Scale bar, 100 μm.

**k** Schematic of primary subcutaneous CT26 colon tumor model establishment and therapeutic regimen.

**l** Gross morphology of tumors in each group. Scale bar, 1 cm.

**m-n** Tumor volume (m) and weight (n) in each group (n = 6).

**o** Tumor inhibition rate in each group (n = 6).

**p** Histopathological evaluation of tumor by H&E staining. Scale bar, 100 μm.

**q** Flow cytometric quantification of PI^+^ dead cells in tumor tissues.

**r** TUNEL and GSDMDC staining of tumor after treatment. Scale bars, 100 μm.

**s** Western blotting analysis of GSDMD cleavage in tumor.

**t** CD8^+^ T cell infiltration level quantified by immunofluorescence. Scale bar, 100 μm.

n.s., not significant. Exact *p* values were calculated using one-way ANOVA with Tukey’s post hoc test (d, j, m-o, t) or Dunnett T3 post hoc test (c, e, h, r).

Figure. S11.

**Supplementary Figure S11**

**PADVs^L@D^ activated the immune response in tumor-draining lymph nodes and tumor microenvironment.**

**a** Fluorescence analysis of PADVs accumulation in tumors and draining lymph nodes. Scale bar, 100 μm.

**b** Ex vivo fluorescence imaging for distribution of different formulations in LNs.

**c** GSDMDC staining of LNs following different treatments. Scale bars, 100 μm (top), 20 μm (bottom).

**d** Western blotting analysis of GSDMD cleavage in LNs.

**e** KEGG enrichment analysis of differentially expressed genes (DEGs) in specific pathway.

**f** Chord diagram visualization of shared DEGs across KEGG enrichment pathways.

**g** Heatmap visualization showing expression patterns of shared DEGs in KEGG enrichment pathways.

**h** Fluorescence images of M2 macrophages (F4/80^+^CD206^+^) and regulatory T cells (CD4^+^Foxp3^+^) in tumors after different treatments. Scale bar, 50 μm.

Figure. S12.

**Supplementary Figure S12**

**PADVs^L@D^-primed immunity showed no cross-reactivity against antigenically distinct tumors.**

**a** Experimental schema of heterologous MC38 tumor rechallenge model following B16F10-primed immune memory.

**b** Gross morphology of MC38 tumors in each group. Scale bar, 1 cm.

**c-d** Tumor volume (c) and weight (d) in each group (n = 6).

**e** Histopathological evaluation of tumor by H&E and TUNEL staining. Scale bars, 100 μm.

**f** Western blotting analysis of GSDMD cleavage in tumor.

**g** Quantification of CD8⁺ T cell infiltration level by immunofluorescence. Scale bar, 100 μm.

**h** Quantification of intratumoral inflammatory factors (n = 6).

n.s., not significant. Exact *p* values were calculated using unpaired two-tailed Student’s t-test (c, d, g, h).

Figure. S13.

**Supplementary Figure S13**

**PADVs^L^ suppress hematogenous tumor metastasis in CT26 tumor-bearing mice.**

**a** Experimental schema of CT26 hematogenous metastasis model establishment and therapeutic regimen.

**b** Gross morphology of primary subcutaneous tumor in each group. Scale bar, 1 cm.

**c-d** The volume (c) and weight (d) of primary tumor in each group at day 21 (n = 6).

**e** Histopathological evaluation of primary tumor by H&E staining. Scale bar, 100 μm.

**f** Longitudinal monitoring of metastatic burden by CT26-luc bioluminescence imaging until day 21 (n = 6).

**g** Gross morphology of lungs with metastatic nodules (yellow arrows), and whole-lung H&E staining with micrometastatic histology (dotted line). Scale bars, 1000 μm (middle), 50 μm (bottom).

**h** Morphometric analysis of metastatic area in lung sections (n = 6).

**i** Quantification of CD8⁺ T cell infiltration level in metastatic tumor foci. Scale bar, 100 μm.

**j** Survival curves of tumor-bearing mice with hematogenous metastasis until day 30 (n = 10).

Exact *p* values were calculated using one-way ANOVA with Tukey’s post hoc test (d) or Dunnett T3 post hoc test (h, i), Mann-Whitney test (c) and Log-rank test (j).

Figure. S14.

**Supplementary Figure S14**

**Systemic biosafety evaluation of PADVs in vivo.**

**a** Schematic diagram of in vivo safety evaluation.

**b-c** Dynamic monitoring of body weight (b) and body temperature (c) in mice during PADVs treatment (n = 6).

**d** Serum biochemical analysis of alanine transaminase (ALT), aspartate aminotransferase (AST), blood urea nitrogen (BUN), creatinine (CREA), creatine kinase (CK) and lactate dehydrogenase (LDH) (n = 6).

**e** Histopathological analysis of major organs by H&E staining. Scale bar, 100 μm.

Table S1.

| **Target** | **Conjugates** | **Manufacture** | **CAT.** | **Application** |
| --- | --- | --- | --- | --- |
| GM130 | / | Santa Cruz Biotechnology | sc-55591 | WB |
| HSP70 | / | Huabio | ET1601-11 | WB |
| Calnexin | / | Abcam | ab22595 | WB |
| integrin-β3 | / | Abcam | ab75872 | WB |
| CCR5 | / | Santa Cruz Biotechnology | sc-17833 | WB |
| CXCR3 | / | Santa Cruz Biotechnology | sc-137140 | WB |
| Na^+^/K^+^-ATPase | / | Abcam | ab76020 | WB |
| F4/80 | / | Cell Signaling Technology | #70076 | WB |
| F4/80 | / | Abcam | ab6640 | IF/TEM |
| CD3 | / | Abcam | ab135372 | WB/TEM |
| GSDMD | / | Abcam | ab219800 | WB |
| CD47 | / | Santa Cruz Biotechnology | sc-53050 | WB/IHC |
| SIRPα | / | Abcam | ab191419 | WB/IF |
| PD-1 | / | Abcam | ab214421 | WB/IF |
| PD-L1 | / | Abcam | ab213480 | WB |
| PD-L1 | / | Servicebio | GB115704 | IHC |
| CD40 | / | Santa Cruz Biotechnology | sc-975 | WB |
| CD86 | / | Santa Cruz Biotechnology | sc-28347 | WB |
| MHC I | / | Santa Cruz Biotechnology | sc-59199 | WB |
| MHC II | / | Santa Cruz Biotechnology | sc-59318 | WB |
| GAPDH | / | YEASEN | 30201ES20 | WB |
| Caspase-11 | / | Abcam | ab180673 | WB |
| HMGB1 | / | Abcam | ab79823 | WB/IF |
| CD3 | FITC | BioLegend | 100203 | FACS |
| CD8a | PE | BioLegend | 100708 | FACS |
| Granzyme B | FITC | BioLegend | 515403 | FACS |
| PD-L1 | PE | BioLegend | 155404 | FACS |
| CD47 | PE | BioLegend | 127508 | FACS |
| SIRPα | FITC | BioLegend | 144006 | FACS |
| PD-1 | FITC | BioLegend | 135213 | FACS |
| F4/80 | PE | BioLegend | 123110 | FACS |
| CD11b | FITC | BioLegend | 101206 | FACS |
| CD11c | APC | BioLegend | 117310 | FACS |
| CD80 | FITC | BioLegend | 104706 | FACS |
| CD86 | PE | BioLegend | 105008 | FACS |
| CD25 | PE | BioLegend | 102008 | FACS |
| CD45 | FITC | BioLegend | 103108 | FACS |
| CD45 | APC | BioLegend | 147708 | FACS |
| CD49b | PE | BioLegend | 108908 | FACS |
| CD3 | APC | BioLegend | 152306 | FACS |
| CD4 | / | Servicebio | GB15064 | IF |
| CD44 | / | Servicebio | GB112054 | IF |
| CD62L | / | Abcam | ab325322 | IF |
| Foxp3 | / | Servicebio | GB150152 | IF |
| F4/80 | / | Servicebio | GB113373 | IF |
| CD206 | / | Servicebio | GB153497 | IF |
| CD45.1 | / | Invitrogen | 14-0453-82 | IF |
| CD11b | / | Abcam | ab184308 | IF |
| CD3 | / | Abcam | ab135372 | IF |
| CD45 | / | Abcam | ab10558 | IF |
| CD31 | / | Invitrogen | PA5-32321 | IF |
| CD8 | / | Abcam | ab217344 | IF |
| GSDMDC | FITC | Santa Cruz Biotechnology | sc-393581 | IF |
| Rabbit Anti-Mouse IgG (H+L) | Alexa Fluor 594 | YEASEN | 33912ES60 | IF |
|  | Alexa Fluor 488 | YEASEN | 33906ES60 | IF |
| Goat Anti-Rabbit IgG (H+L) | FITC | YEASEN | 33107ES60 | IF |
|  | Alexa Fluor 594 | YEASEN | 33112ES60 | IF |
| Mouse Anti-Rat IgG | 15-nm Gold nanoparticles | Solarbio | K0032M | IEM |
| Mouse Anti-Rabbit IgG | 35-nm Gold nanoparticles | Solarbio | K0034M | IEM |

**Table S1**. Antibodies used in this study.
